# Video reconstruction from a single motion blurred image using learned dynamic phase coding

**DOI:** 10.1038/s41598-023-40297-0

**Published:** 2023-08-21

**Authors:** Erez Yosef, Shay Elmalem, Raja Giryes

**Affiliations:** 1 Tel-Aviv University, Tel Aviv-Yafo, Israel; 2 Present Address: Weizmann Institute of Science, Rehovot, Israel

**Keywords:** Electrical and electronic engineering, Imaging and sensing

## Abstract

Video reconstruction from a single motion-blurred image is a challenging problem, which can enhance the capabilities of existing cameras. Recently, several works addressed this task using conventional imaging and deep learning. Yet, such purely digital methods are inherently limited, due to direction ambiguity and noise sensitivity. Some works attempt to address these limitations with non-conventional image sensors, however, such sensors are extremely rare and expensive. To circumvent these limitations by simpler means, we propose a hybrid optical-digital method for video reconstruction that requires only simple modifications to existing optical systems. We use learned dynamic phase-coding in the lens aperture during image acquisition to encode motion trajectories, which serve as prior information for the video reconstruction process. The proposed computational camera generates a sharp frame burst of the scene at various frame rates from a single coded motion-blurred image, using an image-to-video convolutional neural network. We present advantages and improved performance compared to existing methods, with both simulations and a real-world camera prototype. We extend our optical coding to video frame interpolation and present robust and improved results for noisy videos.

## Introduction

Modern cameras are required to satisfy two conflicting requirements: to provide excellent imaging performance while decreasing the space and weight of the system. To address this inherent contradiction, novel design methods attempt to harness fundamental imaging limitations and leverage them as a design advantage. One such example is motion blur, which is a known limitation in photography of dynamic scenes. It is caused due to objects’ movements during exposure, whose duration is set according to lighting conditions and noise requirements. As most scenes are dynamic, light from moving objects is accumulated by the sensor in several consecutive pixels along their trajectory, resulting in image blur. Although blur is an undesirable effect, in this work, we use it for video generation from a single image.

In contrast to motion deblurring methods that aim at sharp image reconstruction, in video generation the goal is to exploit this ’artifact’ for reconstructing a sharp video frame burst that represents the scene at different times during acquisition. Yet, as signal averaging in the acquisition process eliminates the motion direction in the captured image, this task is highly ill-posed. The pioneering work of Jin et al.^1^ suggests a pairwise frames order invariant loss to mitigate this ambiguity. Following works presented solutions using a recurrent video autoencoder network^2^ and a cascaded generator^3^. Yet, as the global motion direction is lost in the acquisition, the processing stage can only assume the direction of the motion for the video reconstruction but cannot really resolve the global direction ambiguity.

To overcome this deficiency, some works suggested capturing multiple frames with different exposures during the acquisition process^4^ or alternatively replacing the sensor with coded two-bucket^5–7^, lensless imaging and rolling shutter effect^8^ or event measurements^9^. Yet, these solutions do not fit with a standard optical system or require capturing multiple images.

A similar task is video-to-video processing, namely, from a blurred and low frame rate video to a sharp and high frame rate video. Such a task can be achieved by applying frame deblurring (such as Ref.^10^) followed by frame interpolation for getting sharp video frames^11,12^. Previous end-to-end methods used either processing conventional camera videos^4,13–16^ or computational imaging methods, such as flutter shutter^17^, coded exposure^18,19^ and event camera^20–22^. More details about related works are presented in the Supplementary Information.

**Figure 1:**
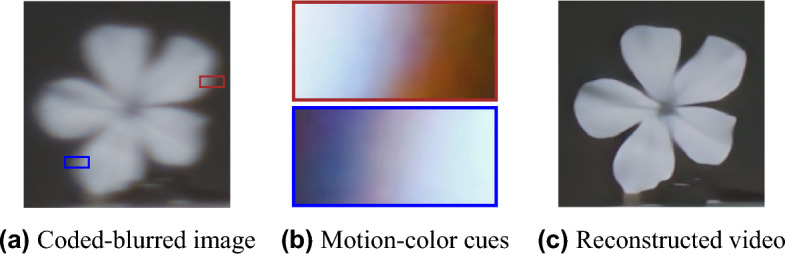
Method demonstration. (**a**) A flower moving left was captured using our dynamic phase-coded camera, which embeds (**b**) color-motion cues in the intermediate image. These cues guide our image-to-video reconstruction CNN, resulting in a (**c**) sharp video of the scene (play the video by clicking on (**c**) in Adobe Reader).

**Figure 2:**
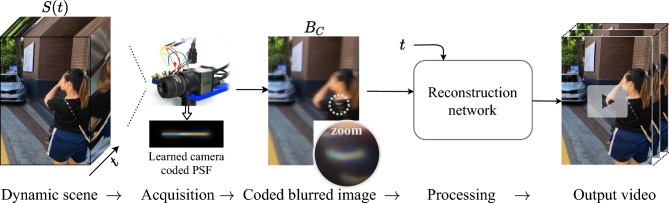
Overview of our suggested method. An acquisition of a dynamic scene using our dynamically phase-coded camera provides an intermediate image $B_C$ which contains scene dynamics cues in its coded motion-blur. We reconstruct sharp video frames of the scene at desired timesteps *t* from the single coded-blurred image $B_C$ using a time-dependent CNN. The optical coding parameters are jointly optimized with the reconstruction network weights using end-to-end learning.

### Contribution

 To overcome the limitations of conventional cameras in dynamic scenes acquisition, we suggest a computational coded-imaging approach (see Figs. 1 and 2) that can be easily integrated in many conventional cameras (equipped with a focusing mechanism) by just adding a phase-mask to their lens (which is a simple process). Joint operation of the phase-mask and focus variation during exposure generates dynamic phase coding, which encodes scene motion information in the intermediate image as chromatic cues. The cues are generated by the PSF nature of our solution (plotted in Fig. 4), which encodes the beginning of the movement by blue and the end by red, e.g., see the zoomed left and right edges of the moving flower in Fig. 1b (enhanced for visualization). These cues serve as guidance to reconstruct a video of the dynamic scene by post-processing the captured coded image (see Fig. 1c).

Our method is capable of generating a sharp frame at any user-controlled time in the exposure interval. Therefore, a video burst at any user-desired frame rate can be produced from a single coded image. The proposed coding and reconstruction approach is based on a learnable imaging layer and a Convolutional Neural Network (CNN), which are jointly end-to-end optimized; the learnable imaging layer simulates the physical image acquisition process by applying the coded spatiotemporal point spread function (PSF), and the CNN reconstructs the sharp frames from the coded image. Our main contributions are:

- A learning based coding method, which requires only a conventional sensor and a lens with a focusing mechanism and is equipped with a simple add-on optical phase-mask.
- The learned code allows using low-power CNNs while achieving high quality video reconstruction from a single image.
- A novel neural architecture that leads to a flexible and modular video from a single image reconstruction, which can be easily adjusted to any desired frame rate video by a simple change in the neural network parameters that do not require re-training.
- End-to-end optimization framework for optical and digital processing parameters for dynamic scene acquisition, which accurately models a spatio-temporal acquisition process.
- Improved video from motion reconstruction, with unambiguous directionality, higher accuracy and lower noise sensitivity, tested in both simulation and real-world experiments.
- A novel video perceptual loss for training neural networks and a metric for video reconstruction evaluation that takes into account both spatial and temporal information.
- A video frame interpolation method that achieves improved results for real-world dynamic scenes using the proposed coding.
- SNR tradeoff analysis for dynamic scenes acquisition and frame interpolation.

## Method

As our goal is to reconstruct video frames from a motion blurred image of the scene, we engineer the camera’s PSF to encode cues in the motion blur of dynamic objects. The coded PSF is achieved using a spatiotemporal dynamic phase coding in the lens aperture, which results in motion-coded blur. The coded blur serves as prior information for the image-to-frames CNN, trained to generate sharp video frames from the coded image. Utilizing the end-to-end optimization ability, the optical coding process is modeled as a layer in the model, and its physical parameters are optimized along with the conventional CNN layers in a supervised manner. The learned optical coding is then implemented in a prototype camera, and images taken using it are processed using the digital processing layers of the CNN.

### Camera dynamic phase coding

Moving objects in a scene during exposure result in motion blur, as the light from a moving object is integrated in different pixels along the motion trajectory. In addition, both static and dynamic objects are blurred by the lens PSF which is never perfect (due to aberrations/diffraction etc). This imaging process is formulated in Eq. (1); the two-dimensional PSF is spatially convolved with the instantaneous scene at any *dt* and integrated during exposure, *B* is the acquired blurred image (all images mentioned are in the linear regime (signal space), i.e. before any non-linear transformations such as gamma correction), *T* is the exposure time, *S*(*t*) and *h* denote the instantaneous sharp scene and the PSF respectively, and $(\underset{Sp}{*})$ denotes the spatial convolution operator (the spatial coordinates are omitted for ease of notation).

1 $$\begin{aligned} B = \frac{1}{T}\int _{0}^{T} \left( h \underset{Sp}{*}\ S(t)\right) \,dt \simeq \frac{1}{N}\sum _{n=1}^{N} \left( h \underset{Sp}{*}S(n)\right) . \end{aligned}$$

The averaging nature of image sensors results in the loss of the motion direction, which introduces inherent ambiguity. Also, as every object moves independently of others, general motion blur is shift-variant. Thus, video reconstruction from undirected motion blur is a highly ill-posed task.

To address both issues, we implement a coded lens designed to embed motion cues in the acquired image, and the prior knowledge about the camera’s time-variant behavior serves as guidance to the reconstruction process of the video burst. We adopt dynamic phase coding in the lens aperture, similar to the motion deblurring method by Elmalem et al.^23^. This method is based on a spatiotemporally coded PSF that encodes motion information in the intermediate image without attenuation of the signal compared to amplitude coding methods^17,24^. Such an approach improves the signal to noise ratio (SNR) compare to amplitude coding methods. The intermediate image is slightly more blurred than a conventional image, but these cues are used to achieve improved reconstruction. Since video reconstruction from a blurred image is a more ill-posed task than deblurring, we improve the imaging method by optimizing the coding parameters for our task using end-to-end learning of them with the reconstruction network.

The camera PSF is generated using a conventional camera equipped with a simple add-on phase-mask; the temporal coding is achieved using a joint operation of the static phase-mask designed to introduce color-focus cues, and a dynamic focus sweep performed during exposure (using a simple focusing mechanism). The phase-mask (originally designed for depth estimation^25^ and extended depth of field imaging^26^) introduces a predesigned chromatic aberration to the lens, generating a controlled dependence between the defocus condition and the color distribution of the PSF. Based on Fourier optics, the PSF of the camera is computed conditioned on the mask specifications, the wavelength and the defocus condition as previous works (extended in Supplementary Information). To get a time-varying PSF the defocus condition (denoted as $\psi$) is changed during exposure, and a temporally coded PSF (denoted as $h(\psi (t))$) is achieved. The instantaneous scene *S*(*t*) is spatially convolved with the corresponding PSF $h(\psi (t))$, resulting in the motion-coded image $B_c$ described in the following formula:

2 $$\begin{aligned} B_c = \frac{1}{T}\int _{0}^{T} \left( h(\psi (t)) \underset{Sp}{*}\ S(t)\right) \,dt \simeq \frac{1}{N}\sum _{n=1}^{N} \left( h(\psi (n)) \underset{Sp}{*}S(n)\right) . \end{aligned}$$

Using the proposed spatiotemporally coded imaging scheme, the dynamics of the scene are encoded in the intermediate image acquired by the camera. Moving objects are smeared in the image with color cues along their trajectories, based on the spatiotemporal PSF $h(\psi (t))$. The acquired coded image is then fed to the reconstruction network trained to decode these cues as guidance for improved video reconstruction. Figure 2 presents these steps visually.

### PSF design

The time varying PSF conditioned on the defocus parameter $\psi$ is computed based on Fourier optics and described in the Supplementary Information. To achieve optimal motion cues encoding in the intermediate image, the imaging process is modeled as a learnable layer (with corresponding forward and backward models using automatic differentiation). The dynamic phase coding acquisition is simulated using the phase-mask characteristics and the focus variation parameters. The defocus parameters are optimized in the end-to-end training process along with the CNN layers, while the phase mask design is fixed (constant) in our setup and described in the Supplementary Information. The initialization method of the optical parameters was tested using different approaches in the acceptable physical range, including linear^23^, random, and even some approaches combined with periodic functions (i.e. sine). The linear initialization produced the best convergence results over all other attempts. Note that as we are using temporal phase coding, we do not change the intensity during the change of the focus and we optimize only the focus as a function of time.

**Figure 3:**
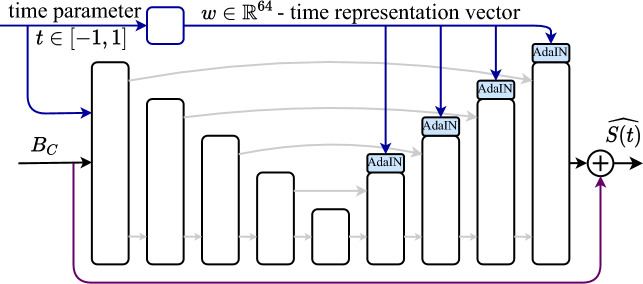
Network architecture. Our CNN is based on the UNet^27^ model, with the coded blurred image and a time parameter as inputs and the sharp reconstructed frame at the output (see Eq. (3)). The decoder part is controlled by the time parameter (using AdaIN^28^), to set the relative time of the reconstructed frame.

### Reconstruction network

Our proposed model for video frames reconstruction from a coded motion-blurred image is based on a single time-dependent convolutional neural network (CNN) with AdaIN mechanism^28^. Our model inputs are the coded-blurred intermediate image and a normalized time parameter $t\in [-1,1]$. The time parameter controls the relative time of the generated sharp frame in the normalized exposure time interval. The output of the model is the estimated sharp scene frame at time *t*, denoted as $\widehat{S(t)}$. Hence, the architecture is designed to reconstruct the scene at any desired instant in the exposure time interval, and thus to create a video at any desired frame rate. We denote it by

3 $$\begin{aligned} \widehat{S(t)} = f(B_c,\,t)\quad t\in [-1,1]. \end{aligned}$$

Our reconstruction CNN (presented in Fig. 3) is based on the UNet architecture^27^, consisting of a four levels encoder-decoder network structure with skip connections between the encoder to the decoder in each level. The double convolution blocks (presented in the original UNet architecture) are improved by adding skip-connections modifying them to the form of dense blocks^29^. The output of the last layer of the model is added to the input image, such that the network learns only the residual correction required to reconstruct the desired frame.

The time parameter is used to reconstruct the frame corresponding to the desired normalized time in the exposure interval. It controls the network by both the AdaIN mechanism^28^ and concatenating it to the input as an additional channel (by expanding the scalar value to image dimensions). To bridge between the shift-invariant convolutional operations of the CNN and the shift-variant (and scene dependent) motion blur that exists in our target application, we leverage positional encoding to add image position dependency to the model. We provide additional details on the architecture and these changes in the following.

#### Positional encoding

**.** Assuming a general scene in which every object might move in a different direction and velocity, an intermediate image captured using our proposed coded lens will contain a shift-variant blur, which is a composition of the color-temporal PSF coding and the spatial movement of the objects. Since convolutions in deep neural networks are shift-equivariant, we added a position dependency to the model to utilize the motion cues, which are assumed to be correlated in the surrounding area that relate to the same object with the same motion characteristics and blurring profile. We adopt Fourier features to get a better representation of the position coordinates^30^. Similar to Metzer et al.^31^, we add a positional dependency to the model by concatenating the Fourier features of the pixel coordinates as additional channels to the input. Five log-linear spaced frequencies $\{w_j\}_{j=1}^{5}$ were sampled in the range [1,20] to generate 20 positional features in total for each pixel coordinate (*u*, *v*) of the image, as presented in previous methods^32,33^. These frequencies are predefined and constant. Each frequency $w_j$ contributes the following four positional features to each pixel using the normalized pixel coordinate in the range of [0,1]:

4 $$\begin{aligned} pos_j(u,v) = \left[cos(w_j\cdot u), sin(w_j\cdot u), cos(w_j\cdot v), sin(w_j\cdot v)\right]. \end{aligned}$$

#### Time encoding

 To achieve a time-dependent CNN, the batch normalization layers in the UNet architecture are replaced with AdaIN layers^28^ controlled by a normalized time parameter. The exposure time interval is normalized to the range of $[-1,1]$ such that $t=0$ corresponds to the middle of exposure time. The time parameter $t\in [-1,1]$ is mapped to a higher dimension vector $w\in \mathbb {R}^{64}$, using an MLP network consisting of two sequential blocks of a linear layer followed by a leaky-ReLU activation function. The encoded time-representation vector *w* is shared across all AdaIN layers and controls the mean and standard deviation of the features in each AdaIN layer.

In each AdaIN layer with an input *x* of *p* feature channels, the new mean $\beta \in \mathbb {R}^{p}$ and standard deviation $\gamma \in \mathbb {R}^{p}$ are obtained from $w\in \mathbb {R}^{64}$ by a two-layer MLP network of the same structure mentioned above. The AdaIN transformation (Eq. (5)) is performed along the features dimension, such that the dimensions of the new mean and standard deviation vectors ($\mathbb {R}^{p}$) are equal to the number of features in each layer. For the normalization step, $\mu (x)$ and $\sigma (x)$ in Eq. (5) are the mean and standard deviation of the input *x*, and computed across the spatial dimensions (according to instance normalization).

5 $$\begin{aligned} \hat{x} = \gamma \cdot \frac{x-\mu (x)}{\sigma (x)} + \beta . \end{aligned}$$

As our scheme is designed to utilize the optically encoded motion cues to generate a sharp frame in a relative time *t*, the encoder part of the UNet is generic, and we apply the temporally controlled AdaIN only on the decoder part of the architecture (as in Fig. 3). We set the encoder part of the UNet model to be time independent by performing instance normalization followed by a learnable affine transformation instead of the AdaIn blocks. In this setting, the encoder is optimized to encode more general information about the image and scene dynamics regardless of the normalized time parameter. The generic encoder and time-specific decoder design enable the network to converge better. Note though that we concatenate the time parameter to the input channels which contain the input image and the positional encoding features. This improves reconstruction performance as shown in the ablation in section in the Supplementary.

#### Dataset

 To train our network and evaluate its performance quantitatively, we used the REDS dataset^34^, consisting of scenes captured at 120 frames per second (FPS). To achieve smoother motion-blur simulation we used $\times 8$ frame interpolation using the DAIN method^11^ (similarly to the process of Nah et al.^34^), to achieve video frames at 960 FPS. Inverse camera response function (CRF) was applied on the frames to convert them from gamma space to signal space, using the inverse CRF transform given with the dataset. To simulate the acquisition of a dynamic scene by our coded camera, the spatiotemporal PSF had been applied to 49 consecutive frames in signal space, which were then averaged along the time axis as in Eq. (2) (where $N=49$). For performance comparison with Jin et al.^1^, conventional camera images were simulated by only averaging the frames without applying the PSF. Due to the applied frame interpolation, not all the 49 frames are true images; therefore only the seven real frames (in indices $n=8k,\; k \in [0,6]$) are used as our GT images for the training/validation/test metrics. For improved generalization, we add additive white Gaussian noise (AWGN) to the simulated blurred images in the signal space, which partially simulates the imaging process noise and improves the robustness of our model and generalization to the camera prototype (different noise levels were set according to the application, as discussed in Experiments section).

#### Loss functions

 We use a linear combination of three losses for the training: pixel-values smooth-L1 loss ($l_{L1}$), perceptual loss ($l_{percep}$) using VGG features^35^, and a video-consistency perceptual loss ($l_{vid}$). Thus, our loss is

6 $$\begin{aligned} l = \alpha _{L1}\cdot l_{L1}+\alpha _{percep}\cdot l_{percep}+\alpha _{vid}\cdot l_{vid}. \end{aligned}$$

The perceptual loss is a known practice for image reconstruction tasks^35^. In this loss, we compute the smooth-L1 distance between the VGG^36^ features of the reconstructed image and the ground truth image.

To improve temporal consistency and perceptuality between consecutive reconstructed video frames, we developed a video loss using a 3D convolution network over the video time-space volume. We use 3D-ResNet^37^, a spatiotemporal convolution network for video action recognition, and compare the network-extracted feature maps between the reconstruction and the GT videos. We used the output of the first three convolution layers of the 3D-ResNet network and averaged the smooth-L1 loss between the features of the ground truth video and the reconstructed video. More details are provided in the Supplementary Information.

## Results

As an experimental validation to our proposed approach, we first train our system (optical coding layer and reconstruction network) and evaluate our results quantitatively (while the optical coding process is simulated), and compare the performance to a previous work^1^. Following the satisfying simulative experiment, we built a prototype camera implementing our spatiotemporal coding and examined our method qualitatively (as pixelwise GT sharp frame bursts are almost impossible to acquire). Lastly, we present an ablation study for our architecture and used methods. Some of the results are presented below, and additional results are presented in the Supplementary and the Video.

### Training details

 We train our model on a training set consisting of 9680 scenes for 40 epochs, with a batch size of 72 samples of patches in size 128×128×3 each. We used Adam optimizer^38^ with learning rate of $10^{-3}$ and weight decay of $10^{-8}$. The loss weightings (as defined in Eq. (6)) are $\alpha _{L1}=1;\;\alpha _{percep}=0.1;\;\alpha _{vid}=0.1$. Additional 2460 scenes are dedicated for validation/testing, such that the quantitative reconstruction performance (simulation section) was evaluated using 1968 scenes dedicated for testing. In the optical coding layer we define a learnable defocus condition vector $\overline{\psi }\in \mathbb {R}^{49}$. We optimize these focus sweep parameters of the camera, which defines the camera time-varying PSF (following the computation presented in Supplementary), and the result obtained following the imaging in Eq. (2). These parameters initialized linearly as discussed in PSF design. To improve robustness we apply flip augmentations and add AWGN to the input image (1% as in Ref.^1^ for simulation, and 3% for our Prototype Camera estimated noise level).

**Figure 4:**
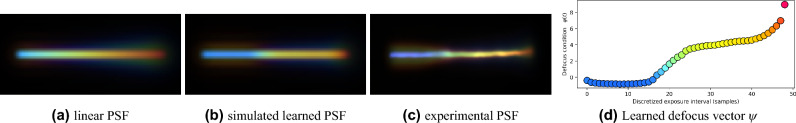
PSF coding. The spatiotemporal PSF coding of the (**a**) linear focus sweep^23^, (**b**) learned focus variation (simulation), and (**c**) same PSF in an experiment. The PSF visualizations represent the blur of a point light source moving horizontally (left to right) during the exposure time. The joint effect of the phase-mask and focus variation during exposure results in different wavelength (color) that is in-/out-of-focus when the point moves. (**d**) The learned parameters of the defocus change during the acquisition interval. The color of each sample represents the color response of the corresponding PSF kernel (at its center) as presented in the Supplementary Information.

The optimized focus sweep parameters (of the imaging simulation layer) presented in Fig. 4d, and resulted in a coding compactly demonstrated in Fig. 4b (the individual PSF kernels are presented in Supplementary). In this example, a motion blur of a white dot moving right is simulated with a coding based on either linear or learned focus sweep (Fig. 4a and b respectively). Compared to the white trace that would have been captured in a conventional camera, the color coding of the motion profile is clearly visible. The learned pattern provides improved coding for video reconstruction, thanks to the end-to-end optimization with the image-to-video CNN. Following the different initialization methods (discussed in PSF design subsection) we infer that a clearly changing code with prominent characteristics is required for the reconstruction, and injective code function (such that each color appears once) helps the reconstruction and provides better results. The learned coding is also validated experimentally on a moving point source (Fig. 4c).

### Simulative experiment

To evaluate the reconstruction results we used a test dataset consisting of motion blurred simulated images (both conventional and coded). We compare our results to the performance of Ref.^1^ that presented a method for video reconstruction from a conventional camera (uncoded) motion blurred images.(The comparison is made only to Ref.^1^ as other related works did not publish their code for evaluation.) We evaluate the models with respect to the GT sharp scene images using PSNR, structural similarity index measure (SSIM)^39^ and our VID metric which we designed to assess a video frame sequence reconstruction quality.

The VID metric uses the output of the first three 3D-convolutional layers of 3D-ResNet network^37^ (similar approach as the video loss), and is computed by taking their average in log scale (in [dB], higher is better, more details are provided in Supplementary). Indeed, the VID metric is similar to the video loss that we use. Yet, we believe that using VID as a performance measure is far due to the following reasons: (i) We observed visually that better VID correlates with improved visual quality, which confirms the use of this loss; (ii) In the same way that it is valid to train a network using a MSE loss and report performance in terms of PSNR, it is valid to use the video loss in our training (which is not the only loss used) and report performance in terms of the VID metric.

**Figure 5:**
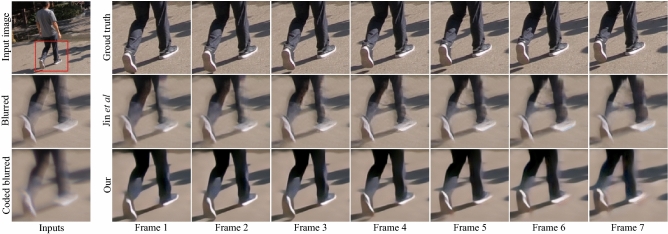
Reconstruction performance (simulation) (top row). GT image and zoom-in for a 7-frames burst, (middle row) conventional blur and Jin et al.^1^ results, and (bottom row) our coded input and reconstruction results. Our method achieves improved results along the entire burst and also provides a higher frame rate video. Click on the blurred input images (left) to play the result videos.

**Figure 6:**
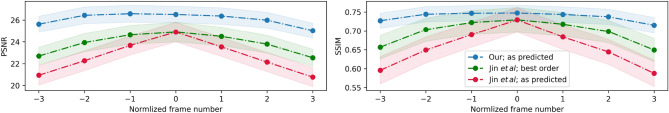
Per-frame performance evaluation. PSNR (left) and SSIM (right) averaged per-frame reconstruction performance for a 7-frames burst, for our method and Jin et al.^1^. Since the motion blur of conventional camera is undirected, we also evaluate the reverse order of Jin et al.^1^ reconstructed frames (compared to the ground truth) for each input scene, and considered the higher results for the ’best order’ presented evaluation.

A visual example of our reconstruction performance is presented in Fig. 5, where improved results along the entire frame burst can be clearly seen. Figure 6 presents the per frame performance in PSNR and SSIM (for a 7-frames burst, as Jin et al.^1^ is limited to such burst length only) averaged over all the test scenes. Table 1 shows the overall statistics of the evaluated metrics of the reconstructions. Since the motion direction is lost in conventional motion blur, the frames’ reconstruction of Jin et al.^1^ may be predicted in the reversed order, i.e. in the opposite motion direction. Thus, each reconstructed scene was compared to the GT in both the predicted order and the reverse order, and the higher one (PSNR-wise) was selected to the ’best order’ average. Note that in $\sim 50\%$ of the cases higher performance is achieved in the reversed order, which shows that the order ambiguity is prominent. Since the coded blur in our camera is designed to provide direction cues, our method is expected to reconstruct the frames in the correct order. Therefore, we do not need to reverse the order for it.

**Figure 7:**
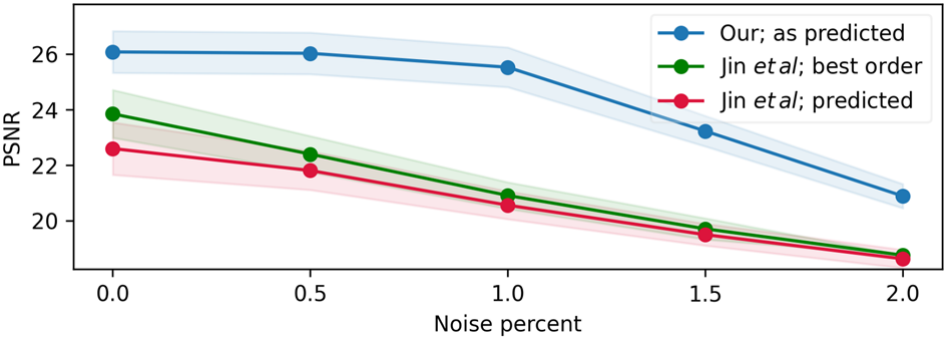
Noise sensitivity analysis. Averaged PSNR results vs. noise level (as percent of the image dynamic range) of our method and^1^ (in both predicted and best order). Our method has better noise robustness, due to the optically embedded cues.

**Table 1:** Quantitative comparison.

	PSNR		SSIM		VID $\uparrow$
Method	Mean	Std	Mean	Std	Mean
Jin et al.^1^	22.6	3.78	0.654	0.143	10.67
Best order, Jin et al.^1^	23.85	3.45	0.69	0.128	10.98
Ours	26.08	3.01	0.737	0.081	11.30

PSNR, SSIM and VID metrics on the entire test set. The PSNR and SSIM metrics averaged over all the reconstruction timesteps during the acquisition interval (7 timesteps), while the VID metric evaluates the whole scene sequence internally.

Comparison of reconstruction quality by our method and Jin et al.^1^ presented both evaluation in predicted order and best order sequence (since the direction ambiguity).

To assess the benefit in noise robustness of the encoded optical cues, a noise sensitivity analysis is carried by evaluating the reconstruction results of our method vs. Jin et al.^1^ for different noise levels (Fig. 7). Similar to the performance analysis in Fig. 6, The reconstruction performance of Jin et al.^1^ is evaluated both in the predicted order and best order. The prominent gap is achieved due to the optically encoded motion information, which allows reconstruction with much better noise robustness.

**Figure 8:**
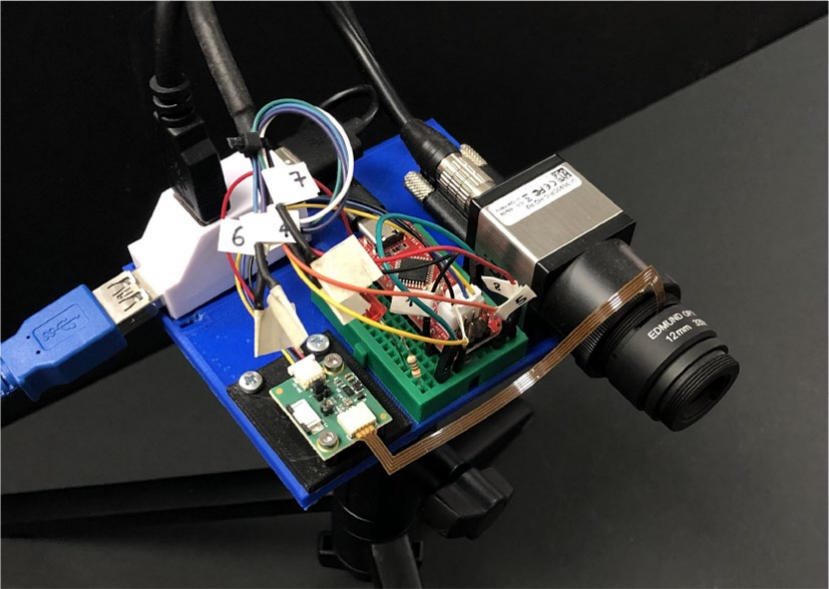
Prototype Camera. the dynamic phase coded camera prototype is based on a commercial camera and a lens with a focusing mechanism, where our phase-mask is incorporated in the lens aperture. The camera flash signal is utilized to trigger the focus variation, controlled using the micro-controller (located near the camera).

### Prototype camera results

**Figure 9:**
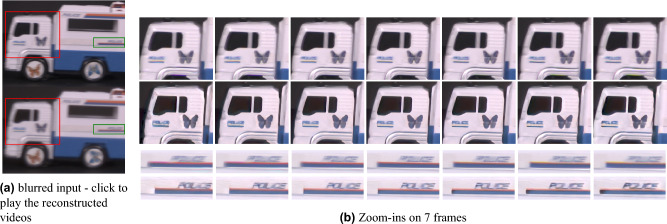
Real-world results. (**a**) blurred image from (top) conventional camera and (bottom) our coded camera; click on the blurred images to play the output videos, (**b**) Zoom-ins on 7 reconstructed frames of (top) Jin et al.^1^ and (bottom) our results. Our method achieves improved results along the entire burst, reconstructs the correct motion direction and also provides a higher frame rate video.

To assess our method on real-world scenes, a prototype camera with dynamic phase coding was implemented. The color-focus phase-mask is incorporated in the lens aperture, and lens defocus setting $\psi (t)$ is set to vary during exposure following the desired learned code using a liquid-lens. The joint operation of the phase-mask and focus variation temporally manipulates the PSF $h(\psi (t))$ as presented in Eq. (2). Our prototype camera (see Fig. 8) is based on a standard C-mount lab-camera (IDS UI-3590CP) equipped with a 4912 x 3684 pixels ($1.25[\mu m]$ pixel pitch) color CMOS sensor^40^. The camera is mounted with a fixed focal length $f=12[mm]$ C-mount lens with a focusing mechanism based on a liquid-lens (Edmund Cx C-mount lens #33-632^41^) and additional details on its design are provided in the Supplementary. Several dynamic scenes had been captured using the prototype camera, and processed using our image-to-frames CNN for different *t* values, thus creating short videos of the moving scenes. For comparison, we took motion-blurred images of the same scenes with a conventional camera (i.e. with constant focus and clear aperture). The results are presented in Figs. 1 and 9. Note how the truck moves and its back wheel rotates (front wheel is fixed) in Fig. 9. Our method provides sharp results and a higher frame rate video. Note also that^1^ reconstructs the motion in the opposite direction.

### Video frame interpolation extension

**Figure 10:**
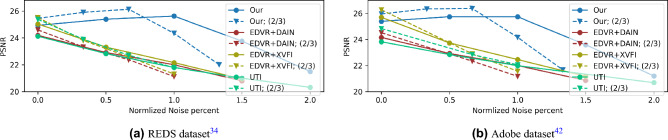
Video frame interpolation - PSNR performance for different noise levels on two datasets. Comparison of PSNR for two exposure intervals: baseline and two-thirds of the baseline. The noise axis was normalized with respect to the exposure interval based on the SNR behavior).

**Figure 11:**
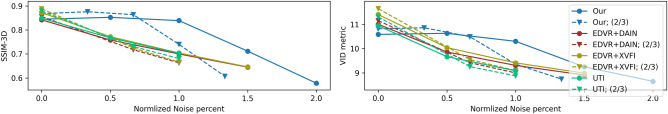
Video frame interpolation - SSIM3D and VID performance for different noise levels on the Adobe dataset^42^. SSIM-3D and VID performance for two exposure intervals: baseline and two-thirds of the baseline. The noise axis was normalized with respect to the exposure interval based on the SNR behavior.

We extend our encoding method for video frame interpolation. Different from the single image task, in this case we use consecutive blurred video frames as input and perform both deblurring and video frame interpolation. Previous works suggested neural networks trained for this task^14,15^, while other works designed solutions for interpolation of sharp input video frames^11,12^. In our method, each frame in the input video is encoded using our learned spatiotemporal PSF. For the sharp frame interpolation methods^11,12^, we first applied a video deblurring algorithm using Wang et al.^10^. We compare the results of the mentioned methods on REDS dataset and Adobe240 dataset^42^, which is a different domain from the data we used for training and validation. We synthesized blurred frames in two timing setups, a baseline exposure and two-thirds of the baseline (see Supplementary). The performances of the methods were evaluated as a function of noise levels by PSNR (Fig. 10a and b) and SSIM-3D and VID (Fig. 11). It is noticeable that our method is more robust and performs better for noisy images, while some of the other methods perform better for clean images which are impractical for real camera images.

The noise effect can be mitigated by using a denoiser at the beginning of the pipeline by applying video denoising prior to deblurring and frame interpolation. We check how much this improves the competing methods in their noise robustness. Specifically, we used VRT^43^, which employs a transformer model for video processing. We used it for denoising with the UTI method and concatenated their denoising and deblurring models for the sharp interpolation methods (DAIN, XVFI). Note that the VRT denoiser is adaptive to noise levels and require this value as input. In addition to applying a denoiser as a pre-processing, we also trained UTI on noisy data with 1% noise added to the conventional images and compared also to it. The results are presented in Fig. 10a and it is noticeable that the drop in performance at large noise levels is smaller compared to Fig. 10. Note that in this figure we average on a smaller test-set (quarter of the frames for each video) since VRT has a very long inference time for inference. We also test Shen et al.^15^ but omit it from the graphs due to low performance (4dB PSNR lower than other methods). Note that since we optimized our model for noise with $\sigma =1$ there is a slight drop in performance for lower noise values. In the Supplementary we elaborate on training details and present additional results.

**Figure 12:**
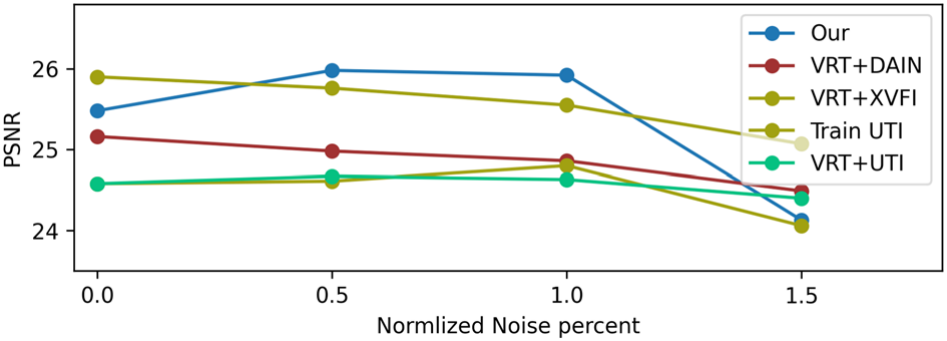
Video frame interpolation with denoising on the REDS dataset^34^. PSNR performance for different noise levels with the two-third baseline exposure setup. A video denoising method, VRT^43^, was applied for denoising and video deblurring to improve the noise robustness of the competing methods. In addition, UTI^14^ is also tested when it is retrained with noisy images with 1% noise level.

### SNR tradeoff in dynamic scenes

While capturing dynamic scenes there is a tradeoff between the signal intensity and motion blur. For lower noise, a long exposure is required and thus a long motion trajectory is obtained in the captured image. On the other hand, a sharp image is obtained by short exposure but the signal level is low, and we get low SNR. Hence, there is a tradeoff between the two, and capturing images/frames without noise is impractical. Thus it is important to have noise robust method for the frame interpolation task. In the noise evaluation figures for the frame interpolation task (Figs. 10a,b and 11), we scaled the noise levels axis of the ”two-thirds” timing setup data compared to the baseline noise levels for the SNR tradeoff evaluation. Namely, since the exposure time in this timing setup is two-thirds of the baseline setup the motion blur is lower and the noise should be higher according to the tradeoff. Such that adding $1.5\%$ noise to short (two-thirds) exposure frames should be compared to $1\%$ noise added to the baseline exposure frames. From the results presented in the mentioned figures we can conclude that for low noise levels (brighter scenes) it will be preferred to use shorter exposure (and get sharper images) while for high noise levels it will be preferred to use longer exposures (Fig. 12).

#### Limitations

 Despite the improved performance achieved, our method still suffers from several limitations. The most prominent are scenes with high-speed or accelerating objects; as our coding method is a composition of the dynamic phase coding and object movement, there is some hidden assumption that this movement (and specifically its acceleration) is not too acute. In such cases, the resulting coded information will be too obscure, with a limited benefit. Even though, our method performs well in a wide variety of cases (as observed in our tests and experiments) since such examples exist in our training dataset. Another limitation relates to the imaging scenario; the temporal part of the coding is focus variation. Therefore, the underlying assumption in such a design is that the entire scene is in the same focus condition (either in- or out-of-focus). Such a design limits our solution to infinite-conjugate lenses (e.g. GoPro cameras). This limitation is more prominent in outdoor scenes with depth where the image is not in the same focus condition. In addition, since our coding is color-based, we assume that objects are not monochromatic, since in such a case the coding ability is degraded. This assumption is acceptable since almost all natural materials are not monochromatic, and practically even some wavelength bandwidth can suffice. Textures are important to indicate a motion in general, and the textures are required to achieve the coded blur in the image for the reconstruction. Even though, if there are no textures the reconstruction becomes a quite trivial task (due to a single color object, where motion and blur are less apparent). Minor artifacts of the model might be observed by a careful analysis of the result videos. The reconstructed result might get smooth since the blurring process may deteriorate the small details in the image, which the model may struggle to recover. Worth noting that since our training dataset is generated using hand-held camera video, there is a camera movement in the synthesized motion blur, and for such small movements, the model learns to reconstruct the motion correctly.

### Inference time comparison

In the image-to-video task, our model reconstructs a sharp frame of the scene in 230ms. The method by Jin et al.^1^ reconstructs 7 frames in 2.45s, namely about 350ms per frame.

In the video-to-video task (frame interpolation), the UTI method^14^ for blurry frame interpolation generate an interpolated frame in 1.1s. The methods for sharp frame interpolation DAIN and XVFI^11,12^ generate a frame in 1s and 2s respectively. Applying video deblurring using EDVR^10^ requires 1.13s per frame prior to the frame interpolation. Using the recent transformers network VRT^43^ for denoising or deblurring prior to the frame interpolation takes 31.5s per frame for each of the tasks. Our video frame interpolation model requires 235ms for each interpolated frame.

Note that our method is faster than the competing methods both in the image-to-video and video-to-video cases. All methods were implemented in PyTorch and tested using Nvidia GeForce GTX TITAN X and only the forward pass on the GPU was monitored.

## Conclusion

A spatiotemporally coded camera for video reconstruction from motion blur is proposed and analyzed. Motivated by the ongoing requirement to improve the imaging capabilities of cameras, the motion blur limitation is utilized as an advantage, to encode motion cues allowing reconstruction of a frame burst from a single coded image. The coding process is performed using a phase-mask and a learnable focus variation, resulting in color-motion cues encoding in the acquired image. This image, along with a relative time parameter *t*, are fed to a CNN trained to reconstruct a sharp frame at time *t* in the exposure time. By choosing a sequence of *t* values, a frame burst of the scene is reconstructed. Simulation and real-world results are presented, with improved performance compared to existing methods based on conventional imaging, both in reconstruction performance and handling the inherent direction ambiguity. Moreover, we present a vast ablation study, noise robustness analysis, learned code contribution (including model size dependency), and central frame performance with a flexibility-quality tradeoff assessment.

An important advantage of our method is that it can assist balancing the various trade-offs that a camera designer has to handle. For example, the promising results achieved hold the potential to extend the method to perform a low-blurred to high-sharp frame rate conversion, achieved with a lower sampling rate and improved light efficiency. This may extend existing photography capabilities with simple and minor hardware changes.

### Supplementary Information

Supplementary Information 1.

Supplementary Information 2.

## Data Availability

The datasets analyzed during the current study are available in the repositories: https://seungjunnah.github.io/Datasets/reds.html and .https://www.cs.ubc.ca/labs/imager/tr/2017/DeepVideoDeblurring/#dataset. Our real-world captures and the trained model are included in this published article (and its Supplementary Information files) for reproducibility.
