# Supplementary material for: Video reconstruction from a single motion blurred image using learned dynamic phase coding
Source: Sci Rep. 2023 Aug 21;13:13625. doi: 10.1038/s41598-023-40297-0 (PMC10442388; doi:10.1038/s41598-023-40297-0)
Supplement: Supplementary file 1 — Supplementary Information 1. [file 41598_2023_40297_MOESM1_ESM.pdf]

Supplementary Information - Video Reconstruction from a Single Motion Blurred Image using Learned Dynamic Phase Coding

Erez Yosef[1,*], Shay Elmalem[1], and Raja Giryes[1]

[1]Tel-Aviv University, Israel
[*]Erez.Yo@gmail.com

ABSTRACT

In this manuscript, we provide supplementary and detailed information on the main paper entitled *Video Reconstruction from a Single Motion Blurred Image using Learned Dynamic Phase Coding*. We describe in more details the related work. We provide more details on the PSF computation, the video loss and metric, and our designed prototype. In addition, we describe and present additional experiments and results that we performed.

1 Related Work

Given a motion blurred image, various methods attempted to reconstruct a sharp image of the scene. Some techniques were developed for the case of conventional imaging and then the reconstruction is only computational, which recently is usually based on training a neural network for this task[1–4]. Other holistic design approaches utilize a computational imaging strategy to encode motion information in an intermediate image and recover the sharp image using corresponding post-processing methods[5–10].

The problem of video reconstruction from a single image takes motion deblurring a step forward by attempting to reconstruct a frame burst of the dynamic scene that resulted in the blurred image, and not only the central sharp frame (see table 1 for an overview). Some works are based on images taken using a conventional camera and apply processing-only methods to obtain a frame burst of the scene. However, without optical coding, this problem is highly ill-posed as even if the edges and textures are reconstructed perfectly, various motion permutations can generate the same motion blurred image (e.g., see Fig. 2 in[11]). Thus, coded imaging approaches were proposed to acquire additional information about scene dynamics and achieve higher quality results.

Conventional imaging based methods. Generating a video sequence from a single motion blurred image is a challenging task: since the temporal order of the reconstructed frames is ambiguous, the problem is highly ill-posed. Jin et al.[11] address the temporal order ambiguity and present a pioneering approach for this task using several reconstruction networks and a novel pairwise frames order-invariant loss. Their suggested method consists of iterative generation of seven sequential frames of the scene, starting from the central frame reconstruction and proceeding to the edge frames of the dynamic scene using the preceding reconstruction results. The method's architecture limits reconstruction to only seven frames of the scene in the exposure interval, and it uses three different trained models for the reconstruction process. Purohit et al.[12] present a solution for video reconstruction using motion representations of the scene learned by a recurrent video autoencoder network. Zhang et al.[13] suggested a detail-aware network using a cascaded generator. The work in[18] proposed a solution for rendering sharp video of a face from new viewpoints from a single motion-blurred image. All of these methods suffer from inherent motion direction

Method	Acquisition	Input	Output size
Computational only[11–13]	conventional	image	fixed
Multiple exposures[14]	short-long-short exposures	3 images	dynamic
Coded two-bucket[15,16]	C2B sensor	two coded images	fixed
Event camera[17]	event sensor	50-100 events vector	dynamic
Proposed method	dynamic phase coding	coded image	dynamic

Table 1. Overview of existing solutions for video reconstruction from a motion-blurred scene.

ambiguity, and their reconstruction performance is more sensitive to noise (as discussed in[19,20] and empirically presented in experiments section of the main paper).

Coded imaging based methods. In order to handle the inherent limitations of conventional imaging, some works adopted computational photography methods for image deblurring and video frames recovery. Raskar et al.[5] introduced an amplitude-coded exposure technique using a fluttered shutter for motion deblurring. This method performs temporal binary amplitude coding, resulting in a wider frequency response, which is utilized for improved motion deblurring results. Levin et al.[6,7] presented a parabolic motion camera with motion invariant PSF utilized for non-blind motion deblurring. Both of these approaches are limited to the reconstruction of a single image.[21] presented a spatial-temporal coding exploiting the rolling shutter of a CMOS sensor. Dynamic phase coding in the lens aperture for motion coding was presented in[22] for motion deblurring. This coding embeds motion cues in the intermediate image for improved deblurring performance. For video restoration from a single coded-blurred image, several approaches had been presented, such as using an event camera[17], or coded two-bucket (C2B) sensor[15,16,23], which both require a non-conventional sensor or lensless imaging and rolling shutter effect[24], which omits the lens and therefore changes the entire imaging concept even for static scenes. We adopt the coding method of[22], which is based on a commercial sensor and lens (with focusing mechanism), equipped with a simple add-on optical element, which allows unambiguous motion cues encoding. Different from[22] that performs only image deblurring, we aim at reconstructing a video of the motion in the scene from a single motion blurred image, which is a vaster and more ill-posed task.

A closely related problem is the reconstruction of a sharp and high frame rate video from motion blurred and low frame rate video, using either processing conventional camera videos[14,25–27] or computational imaging methods[28–31]. These methods require video input (which solves the direction ambiguity problem) and are not applicable to single image input.

Deep optics. As the end-to-end backpropagation-based optimization process of deep models proved itself to be very efficient for various tasks, its power was also harnessed for optical design, either for a standalone optical system design process or jointly with a post-processing algorithm (for recent review on this topics see[32,33]). Specifically for enhanced optical imaging applications, this scheme had been presented for extended depth of field[34–37], depth estimation[38–41], high dynamic range[42,43], ray tracing[44,45], other tasks[46–50] and several microscopy applications[51–54], to name a few. Yet, it was not considered for the problem of video from blur.

2 PSF computation

The PSF of the camera which is conditioned on the time varying defocus parameter (denoted as $h(\psi(t))$) is computed numerically to simulate the camera acquisition process (presented in the main paper), and for the end-to-end training of the defocus condition parameters vector $\overline{\psi} \in \mathbb{R}^{49}$ (experiment section of the main paper). The computation is performed according to Fourier optics, as used also in[34,38]. In out-of-focus imaging the ψ defocus measure is defined as:

$$\psi = \frac{\pi R^2}{\lambda}\left(\frac{1}{z_o} + \frac{1}{z_{img}} - \frac{1}{f}\right) = \frac{\pi R^2}{\lambda}\left(\frac{1}{z_{img}} - \frac{1}{z_i}\right), \tag{1}$$

where f is the focal length, R is the exit pupil radius, λ is the illumination wavelength, z_{img} is the sensor plane, and z_i is the ideal image plane for an object located at z_o. The in-focus circular pupil function is denoated as $P(\rho,\theta)$. By adding a coded pattern (amplitude, phase or both) at the exit pupil, the PSF of the system can be manipulated by a pre-designed pattern. The coding phase mask located at the aperture is denoted as $C(\rho,\theta)$, which is a circularly symmetric piece-wise constant function representing the mask phase shift rings. Such that, for each ring k between $r_{k1} < \rho < r_{k2}$ it holds that $C(\rho,\theta) = exp\{j\phi_k\}$ where ϕ_k is the phase shift of the ring. The specific parameters presented in section 4 The defocus parameter ψ measures the maximum quadratic phase error at the aperture edge, such that we get:

$$P_C(\psi) = P(\rho,\theta)\cdot C(\rho,\theta)\cdot exp(j\psi\rho^2) \tag{2}$$

Following[55], the PSF of an incoherent imaging system is defined as:

$$h(\psi) = |\mathscr{F}\{P_C(\rho,\theta;\,\psi)\}|^2, \tag{3}$$

where $\mathscr{F}$ denote Fourier transform.

We compute the PSF for the RGB colors ($\lambda \in \{610, 535, 455\}nm$) to simulate the camera acquisition using RGB images as described in the main paper. It is an approximation of the real imaging system which applies a specific PSF for each wavelength in the full spectrum of light. Under the assumption that the PSF changes slowly in λ compared to the bandwidth of the color filter array of the camera (Bayer filter, for each color of RGB) the approximation holds.

The PSF is a two-dimensional continuous function in the spatial coordinates of the image. Due to the focus change during the exposure ψ changes and thus the PSF changes continuously in time. For the acquisition simulation (described in the main paper) the spatio-temporal PSF was discretized in time and space as presented in fig. 2.

3 Video loss and video metric

For a video loss and video metric (for train and evaluation respectively) we used 18-layers ResNet3D model[56] that performs 3D spatiotemporal convolutions on video time-space volume. We use the outputs of the three first convolution layers (namely `conv1`, `conv2` and `conv3`) for the reconstructed frame sequence and the ground truth frames (consist of 7 frames each as described in the network section of the main paper). We compute Smooth L1 loss between the two features for each layer output, such that we get three scalar values. These values (denoted as l_k) represent the similarity both in spatial and temporal dimensions. For training, we average the three components to a single loss value (denoted as l_{vid} in the network section of the main paper). For the video sequence evaluation using the video metric, we compute each of the three components in log scale (same as PSNR) and averaged them to a single value (denoted as **VID**)

$$VID_k = -10 \cdot \log_{10} l_k \tag{4}$$

$$VID = mean(VID_1, VID_2, VID_3) \tag{5}$$

4 Dynamic phase-coded camera prototype

Our method is based on a dynamic phase coded camera, designed to embed color-motion cues in the intermediate image, and a corresponding CNN trained to decode these cues and reconstruct a sharp frame burst. After achieving satisfying simulation results (i.e. with simulated coded images), we assembled a prototype camera implementing our proposed dynamic phase-coding. As mentioned in the paper, our coding method is relatively simple and based mostly on conventional commercial parts. As such, it can be easily integrated into any camera equipped with a focusing mechanism.

The coding is achieved jointly using a phase-mask in the lens aperture and by performing a focus sweep during the exposure time. Following the work in[22], we use a similar phase-mask, comprised of two phase rings. The phase-mask aperture diameter is $D = 2.3[mm]$; the first ring (inner-to-outer) radii are $r = [0.633\ 0.92]mm$ and its phase shift is $\phi = 6.5[rad]$; the second ring radii are $r = [0.92\ 1.15]mm$ and its phase shift is $\phi = 13.2[rad]$ (the phase-shifts are measured with respect to $\lambda = 455[nm]$, which is the peak wavelength of the camera's blue channel). The phase-mask is fabricated using conventional photo-lithography and wet etching process.

The dynamic PSF encoding is achieved by applying the learned focus variation during the exposure time. The focus change is performed electronically, using the camera focusing mechanism, controlled by a dedicated micro-controller[57]. The micro-controller contains the learned focus sweep parameters, and triggers the required coding in synchronization with the exposure (utilizing the camera flash-signal, designed to indicate the start of the exposure). Note that although various components had been used in our implementation, the coding can also be implemented easily on existing cameras (assuming the availability of API to the focusing and exposure mechanisms).

5 Additional experiments and models architectures

5.1 Deblurring models

For the deblurring task, we train the same model architecture both for the linear code and the learned code (presented in table 3). We used the proposed Unet model with a few minor adaptations due to the different task. Since the time parameter is irrelevant (always $t = 0$) we did not concatenate a time channel to the input image. moreover, we replace AdaIN with group normalization since it is more stable during training, and set the groups number to 16.

5.2 AdaGN instead of AdaIN video model

In Table 2 we present a model with adaptive group normalization instead of AdaIN (config-g). In this architecture, each instance normalization was replaced using group normalization with 16 groups. Following the normalization we perform an affine transform, the same as performed in AdaIN.

5.3 Degraded architectures

For the degraded models presented in fig. 4 we used Unet architecture[59] in depth of 2 for the encoder and the decoder parts (two down/up-sampling), and with Mobile-Net convolution blocks[60]. We added a skip connection from the input to the output, and

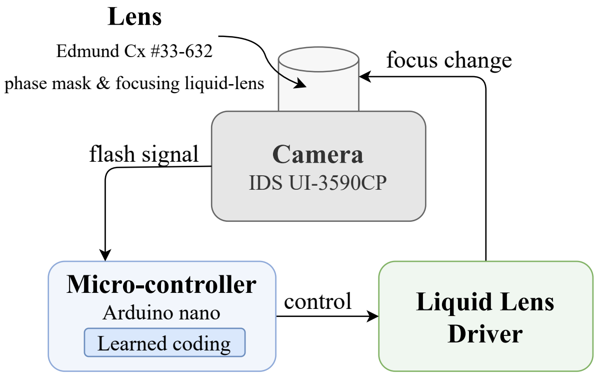

Figure 1. Prototype Camera Diagram. The flash signal from the camera initiates the learned focus variation during the exposure using a micro-controller, such that the designed dynamic phase coding is performed and a motion-coded image is acquired.

neither added the time concatenated channel nor the positional encoding. for B2D2 architecture we used double convolution blocks, while for B1D2 we used a single convolution block. each convolution consist of Inverted-Residual block[60] followed by Leaky-Relu activation. the batch normalization in the Inverted-Residual block was replaced by AdaGN as described before. all the rest details remain the same as the proposed architecture.

5.4 Ablation Study

We apply an ablation study for the proposed method and architecture to evaluate the contribution of each component in our system. table 2 presents the different experiments applied and tested configurations, and table 3 presents the different coding and architectures contributions. Presented in table 2, firstly we started from a UNet architecture controlled by a time parameter using AdaIN modules as described in the network section of the main paper, where the input is the blurred image only, and the output is the sharp frame in the desired relative time in the exposure interval, and trained without the video-perceptual loss. Keeping the encoder part of the UNet uncontrolled by the time parameter (using instance normalization instead of AdaIN) enables better reconstruction results (config-a in table 2) compared to the full AdaIN network, both in encoder and decoder (config-0 in table 2). The following configurations include the addition of image-coordinates positional encoding features (config-b) and the time parameter concatenated to the input image (config-c). These features achieve improvement in PSNR while the similarity measure is slightly decreased, however, while testing the models on the prototype camera images we noticed better generalization to the real world images using these additions. Adding the video-frames perceptual loss (by setting $\alpha_{vid} = 0.1$, see config-d) we get improvement both in PSNR and SSIM. To comprehend the improvement of our optics and computational imaging method for the task, we train our best network (config-d) on uncoded images (i.e. temporal averaging only), and evaluated the results (config-e). Without the phase coding we observe a significant performance degradation, which validates the optical coding benefit to the reconstruction ability. Using the learned temporal coding we gain improvement in both reconstruction metrics (config-f), and we consider it as our proposed model. From the VID metric evaluation, which represents the video reconstruction perceptuality, we observe significant improvement using our learned coding compare to the linear (config-f and config-d respectively)

To comprehend better the improvement achieved by our learned code (compare to the linear coding) we evaluate different size models as presented in fig. 4. Using smaller models (e.g. in limited resources conditions) the improvement of the learned code becomes more significant and it contributes to better reconstruction results of the degraded models. We conclude that while a large network is capable to solve the harder task (linear code), for a smaller (and weaker) network the learned code is more meaningful to achieve better results. For the smaller networks, we used U-net with encoding depth of two levels and Mobile-Net blocks[60] (additional details about the models' architecture described in section 5).

In table 3 we evaluate the coding methods contribution by the central frame performance (namely the deblurring performance). The uncoded exposure using[11] reconstruction method achieves inferior results. The performance of the naive UNet architecture with linear coding is presented as well. The suggested model is also presented in the table. It is noticeable that the improved UNet improves the results while the learned PSF achieves additional improvement. We also trained our Unet model only on the central frame (as a deblurring task) to infer about the flexibility-quality tradeoff. we used the linear code (equivalent to[22]) and our learned code. Even though our proposed method suffers from a small performance drop on the middle frame, it allows the flexibility of generating a video sequence of the scene with any desired number of frames. For these models we

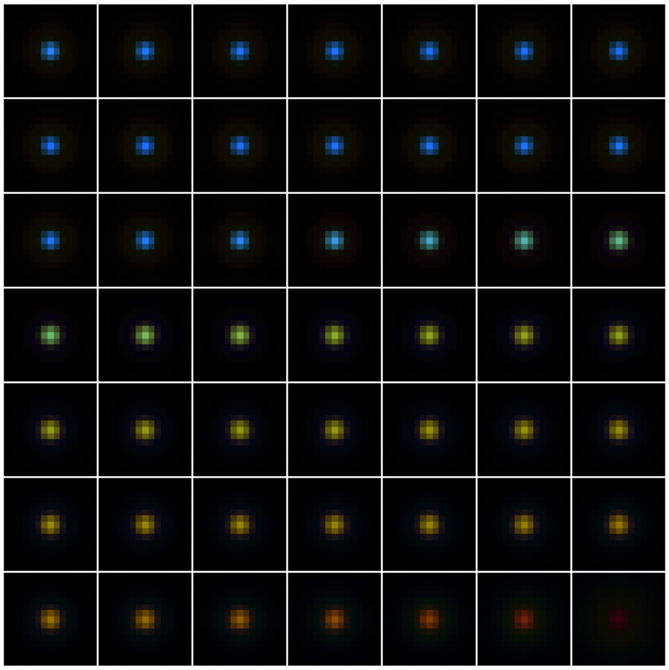

Figure 2. Learned PSF kernels. The time-variant camera PSF represented by 49 RGB color kernels, starts at the upper-left kernel (blue) and ends at the bottom-right kernel (red) in row-major order. These are a simulation of the camera PSF, computed as discussed in section 2

replaced AdaIN with group normalization (GN) for better stability (additional details in section 5). We also present another variant of our model using AdaGN instead of AdaIN (config-g in table 2), such a model achieves better results for PSNR and SSIM, but similar results in VID metric (architecture details in section 5. Even though, we consider config-f in table 2 as our suggested model for the comparisons and the obtained visual results.

5.5 VID Loss/Metric Validation

In this section we validate the proposed video loss and present the spatial and temporal consistency preserving behavior of the metric. Since the loss is based on a pretrained 3D-ResNet architecture, the explainability of the model is a challenging task. The video loss is reference-based, namely, it requires the ground-truth video along with the distorted (reconstructed) video to compare the deep features difference. The well known MSE loss is also reference-based in this sense, but it compares the pixel values pixel-wise. The MSE loss has neither spatial nor temporal dependencies between pixels, unlike our video loss.

The first observation is that both losses typically decrease or increase together depending on the similarity between the distorted and ground-truth videos. To present the spatio-temporal consistency of the video loss we use the following test scheme: we choose a fraction p of pixels of the video to be distorted. Note that for $p = 1$ we get the worst loss value since all

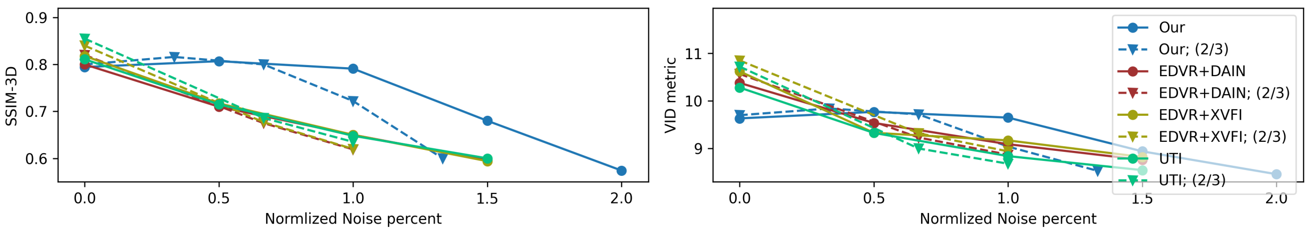

Figure 3. Video frame interpolation - SSIM3D and VID performance for different noise levels on REDS dataset[58]
Comparison of SSIM-3D metric and our VID metric for two exposure intervals: baseline and two-thirds of the baseline. The noise axis was normalized with respect to the exposure interval based on the SNR behavior (more information is described in the SNR section of the main paper).

	Method	PSNR ↑	SSIM ↑	VID ↑
(0)	time dependent encoder	21.5	0.69	10.59
(a)	UNet	25.06	0.73	11.07
(b)	+ positional encoding	25.16	0.728	11.08
(c)	+ time concatenation	25.70	0.705	11.15
(d)	+ video perceptual loss	25.93	0.735	11.12
(e)	(d) w/o phase coding	22.96	0.645	10.57
(f)	(d) with learned PSF	26.08	0.737	**11.30**
(g)	+AdaGN instead of AdaIN	**26.38**	**0.75**	**11.30**

Table 2. Ablation study. To assess the contribution of each feature of our method, we performed a gradual performance evaluation. The PSNR and SSIM metrics were averaged over all the reconstruction timesteps during the acquisition interval, while the VID metric evaluates the whole scene sequence internally. All the metrics were averaged over the test set scenes.

Method	PSNR ↑	SSIM ↑
Uncoded[11]	24.9	0.729
UNet + linear code (a)	25.48	0.74
Suggested UNet + linear code (d)	26.3	0.744
Suggested UNet + learned code (f)	26.51	0.747
Suggested UNet + AdaGN + learned code (g)	**26.75**	**0.759**
Deblurring models (trained only on the central frame)		
Linear coed - central frame with GN	26.73	0.745
Learned coed - central frame with GN	**27.05**	0.757

Table 3. Central Frame Performance. Averaged PSNR/SSIM metrics on the central frame (on the test dataset) for different coding methods: uncoded, linear and learned. (the letter in the parenthesis indicates the entry in table 2). We also present models trained only for the deblurring task (central frame reconstruction) for flexibility-quality tradeoff assessment.

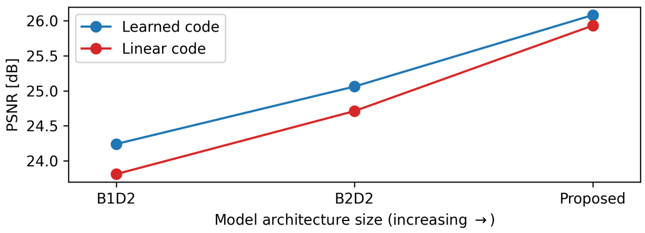

Figure 4. Reconstruction PSNR vs. model size. PSNR reconstruction results for three different size models: Our proposed model and two lighter Unet models with encoding depth of two and double/single convolutions block (B2D2 and B1D2 respectively, details in section 5). Our learned code improvement is more significant as the model is more degraded and less powerful.

Metric	Inconsistent		Consistent		Loss change	
	spat	temp	spat	temp	spat	temp
Frame blur:						
MSE loss (10^{-4})	6.65	6.68	6.9	6.68	+3.53%	∼0%
VID loss (10^{-2})	3.92	4.49	3.38	4.1	**-16.1%**	**-10.2%**
Frame shift:						
MSE loss (10^{-3})	8.22	8.21	8.54	8.19	+3.83%	∼0%
VID loss (10^{-2})	10.9	10	7.36	8.4	**-47.7%**	**-18.4%**

Table 4. Consistency significance for VID loss we tested the spatial/temporal consistency (denoted as spat./temp. respectively) effect on the VID and MSE losses (for $p = 0.5$). The VID loss is very affected by the consistency of the distorted data, and thus encourages the models for consistent predictions.

the video pixels are distorted, and for $p = 0$ we get zero loss (identical to GT). We choose the pixels to distort in three methods: (i)random over space and time; (ii) not altering some spatial blocks that preserve spatial consistency (a rectangle in each frame randomly located over the time axis); and (iii) not altering some spatial-temporal blocks that preserves both a consistency in time and space (rectangle in each frame in the same location over time axis). The results are presented in fig. 5. It is noticeable that for the spatio-temporal consistent distorted video the video loss gives the lowest results (for each p value). Due to MSE loss's lack of spatial and temporal dependence, the metric behaves the same regardless of the pixel sampling (and distortion) method.

For an additional observation, we fixed $p = 0.5$ and tested the temporal and spatial consistency for two video distortion types: spatial Gaussian blur ($\sigma = 1$) and pixels shift (3 pixels in both axes). For the inconsistent temporal/spatial case, we set every other frame/row to be distorted, while for the consistent case we set the first half of the frames/rows to be distorted. As presented in table 4, the VID loss is much better in the consistent case (for both spatial and temporal), while the MSE loss is not affected much due to a lack of axial correlation. Hence, the VID loss encourages spatial and temporal consistency as a training loss.

6 Video Frame Interpolation Details

For video frame interpolation model we used three consecutive coded blurred frames as an input to the model, and reconstruct a single frame in time interval $t \in [-1, 1]$. The other details of training remain as in the image-to-video case. We set two video timing setups for the training and evaluation. Using 960 fps sharp video data set we generated "48-8" blurred video data by averaging 48 frames in linear space to a single blurred frame (equivalent to 50ms exposure), and passing 8 frames as reset time of the camera (intervals of 56 frames total). This timing setup is considered as our baseline. In the second timing setup, denoted as two-thirds of the baseline, we generate "32-16" dataset accordingly. The exposure interval is 33.3ms in this timing setup, which is two-thirds of the baseline. On training, we use batches of both timing setups to generalize the reconstruction for the different timings. Note that the t parameter represents both intra- and inter-blur frame reconstruction for the specified interval, i.e. a sharp frame which is part of the blurred input image, and a sharp frame that is between blurred frames (the camera reset time).

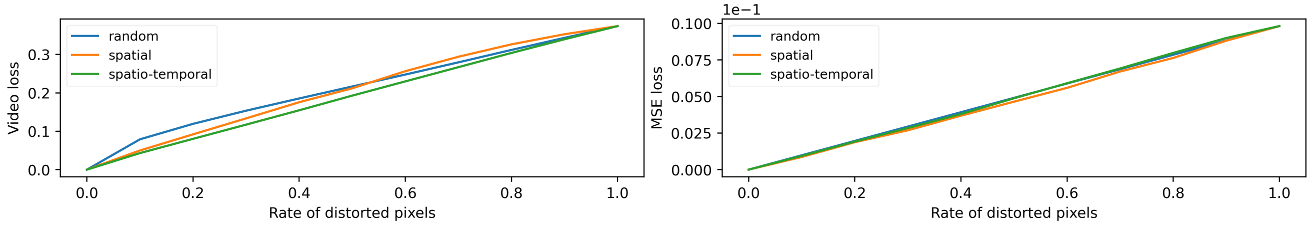

Figure 5. Video loss validation by consistent distortions. For the VID loss it is noticeable that the spatio-temporal consistency distortions achieve the lower loss for all distortions rates, and the loss is affected by video consistency. On the other hand, MSE loss has no spatial correlation and thus is not affected by the spatio-temporal consistency.

6.1 Adobe240 Dataset

To verify our method on another dataset than the one we used for training and validation, we tested the models on the Adobe240 dataset[61] and presented our results in the main paper. Since this dataset is oriented for hand-held camera video deblurring, we picked 10 videos of dynamic scenes with less camera shake for our evaluation. Same as was done for REDS dataset, before creating the blurred frames by averaging consecutive frames we perform frame interpolation by a factor of 4 using[62] to get 960fps video. Inverse CRF was applied on the frame prior to the PSF convolution and temporal averaging (blurring) for blurred video simulation.

6.2 Prototype Camera Video Acquisition

We captured videos using our prototype camera with the temporal phase coding for each frame. We follow the baseline timing, namely 6/7 of the cycle is exposure time and 1/7 is for reset time. We capture in 4 fps, and the exposure time was set to $\sim$214ms accordingly. the reset time in this case is 35.7ms which is enough for the liquid lens to return to the initial state for the next frame capture. Even though high pfs was not our goal for the prototyping, the more prominent bottleneck in our case was the data transfer of the camera due to high-resolution frames acquisition (3200x2400) and not the liquid lens. As we did in the single image models, for the prototype reconstructions we trained a model with 3% noise since real world images are noisier than the 1% noise used for evaluation and comparisons. We placed colourful images on a rotating wheel to control the rotation speed. the images are under free use creative commons license from "pixnio" website. The captured blurred videos and the reconstructed videos are presented in the supplementary material.

7 Additional Image-to-Video Results

In addition to the image-to-video results presented in the paper, we present additional results in the supplementary video and here. The reconstructed videos were generated with 25 frames since we can choose any frames number using our time dependent CNN. The frame-rate difference compared to[11] (which is limited to 7-frames only) is clearly noticeable in the supplementary video. A comparison between the frames in our results and[11] are presented in fig. 6, fig. 7 and fig. 9. Our method achieves improved results along the entire frame burst.

References

1. Zhang, J. *et al.* Dynamic scene deblurring using spatially variant recurrent neural networks. In *2018 IEEE/CVF Conference on Computer Vision and Pattern Recognition*, 2521–2529, DOI: 10.1109/CVPR.2018.00267 (2018).
2. Tao, X., Gao, H., Shen, X., Wang, J. & Jia, J. Scale-recurrent network for deep image deblurring. In *IEEE Conference on Computer Vision and Pattern Recognition (CVPR)* (2018).
3. Kupyn, O., Budzan, V., Mykhailych, M., Mishkin, D. & Matas, J. Deblurgan: Blind motion deblurring using conditional adversarial networks. In *IEEE Conference on Computer Vision and Pattern Recognition (CVPR)* (2018).
4. Nah, S., Kim, T. H. & Lee, K. M. Deep multi-scale convolutional neural network for dynamic scene deblurring. In *The IEEE Conference on Computer Vision and Pattern Recognition (CVPR)* (2017).
5. Raskar, R., Agrawal, A. K. & Tumblin, J. Coded exposure photography: motion deblurring using fluttered shutter. *ACM Trans. Graph.* **25**, 795–804 (2006).
6. Levin, A., Sand, P., Cho, T. S., Durand, F. & Freeman, W. T. Motion-invariant photography. *ACM Transactions on Graph. (SIGGRAPH)* (2008).

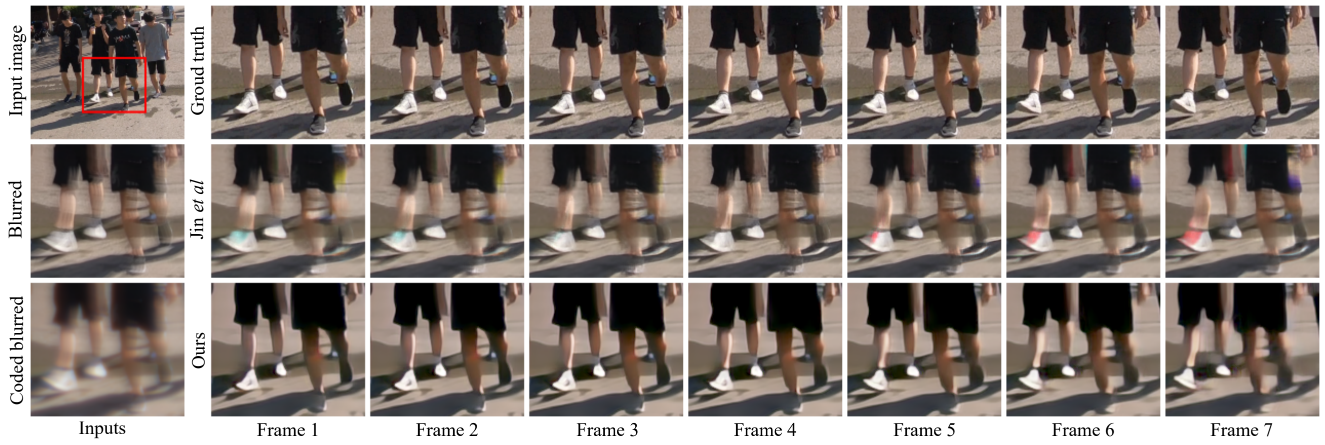

Figure 6. Reconstruction performance (simulation) for seven frames. (top row) GT image and zoom-in for a 7-frames burst, (middle row) conventional blur and Jin et al.[11] results, and (bottom row) our coded input and reconstruction results. The full result videos are presented in the supplementary video.

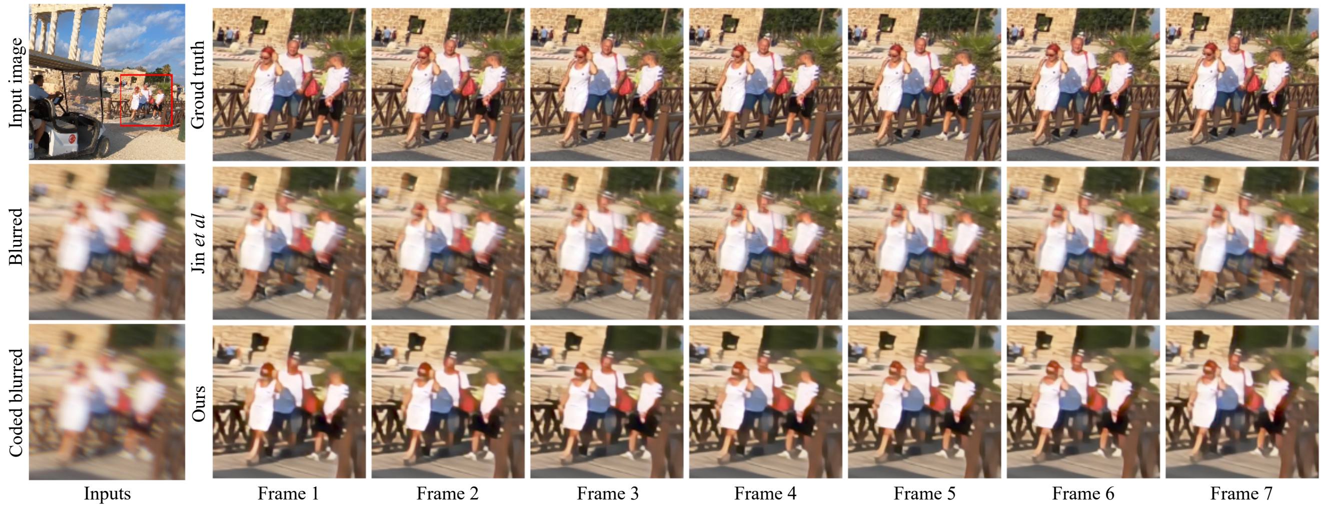

Figure 7. Reconstruction performance (simulation) for seven frames. (top row) GT image and zoom-in for a 7-frames burst, (middle row) conventional blur and Jin et al.[11] results, and (bottom row) our coded input and reconstruction results. The full result videos are presented in the supplementary video.

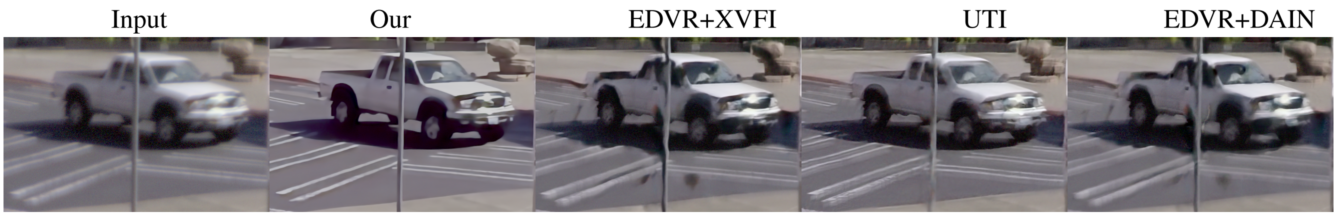

Figure 8. Frame interpolation results. For synthesized blurred input from Adobe240 dataset with noise $\sigma = 0.5$ and the "48-8" timing setup. Left to right: Input blurred frame, our reconstruction sharp frame, and three alternative methods using a conventional camera.

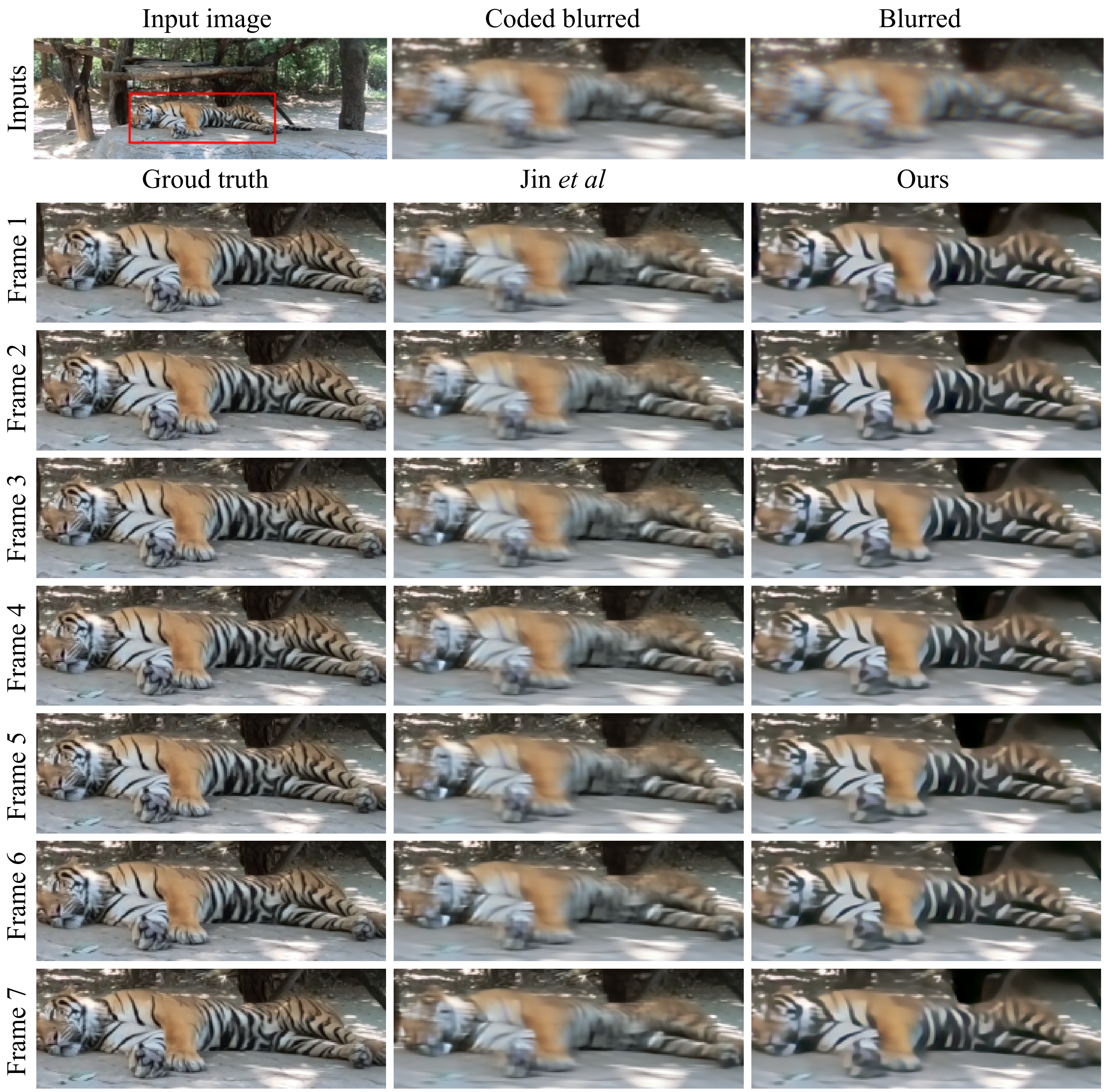

Figure 9. Reconstruction performance (simulation) for seven frames. (left column) GT image and zoom-in for a 7-frames burst, (middle column) conventional blur and Jin et al.[11] results, and (right column) our coded input and reconstruction results. The full result videos are presented in the supplementary video.

7. Cho, T. S., Levin, A., Durand, F. & Freeman, W. T. Motion blur removal with orthogonal parabolic exposures. In *IEEE International Conference on Computational Photography (ICCP)*, 1–8 (2010).

8. Ben-Ezra, M. & Nayar, S. Motion Deblurring using Hybrid Imaging. In *IEEE Conference on Computer Vision and Pattern Recognition (CVPR)*, 657–664 (2003).

9. Srinivasan, P. P., Ng, R. & Ramamoorthi, R. Light field blind motion deblurring. In *Proceedings of the IEEE Conference on Computer Vision and Pattern Recognition (CVPR)* (2017).

10. Mohan, M. M. R., Rajagopalan, A. N. & Seetharaman, G. Going unconstrained with rolling shutter deblurring. In *IEEE International Conference on Computer Vision (ICCV)* (2017).

11. Jin, M., Meishvili, G. & Favaro, P. Learning to extract a video sequence from a single motion-blurred image. In *IEEE Conference on Computer Vision and Pattern Recognition (CVPR)* (2018).

12. Purohit, K., Shah, A. B. & Rajagopalan, A. N. Bringing alive blurred moments. In *IEEE/CVF Conference on Computer Vision and Pattern Recognition (CVPR)*, 6823–6832 (2019).

13. Zhang, K. *et al.* Every moment matters: Detail-aware networks to bring a blurry image alive. *Proc. 28th ACM Int. Conf. on Multimed.* (2020).

14. Rengarajan, V. *et al.* Photosequencing of motion blur using short and long exposures. In *IEEE/CVF Conference on Computer Vision and Pattern Recognition Workshops (CVPRW)*, 2150–2159 (2020).

15. Shedligeri, P., Pal, A. & Mitra, K. Video reconstruction by spatio-temporal fusion of blurred-coded image pair. In *2020 25th International Conference on Pattern Recognition (ICPR)*, 7953–7960, DOI: 10.1109/ICPR48806.2021.9412968 (IEEE Computer Society, Los Alamitos, CA, USA, 2021).

16. Shedligeri, P., S, A. & Mitra, K. A unified framework for compressive video recovery from coded exposure techniques (2020). 2011.05532.

17. Pan, L. *et al.* Bringing a blurry frame alive at high frame-rate with an event camera. In *Proceedings of the IEEE/CVF Conference on Computer Vision and Pattern Recognition (CVPR)* (2019).

18. Meishvili, G., Szabó, A., Jenni, S. & Favaro, P. Learning to deblur and rotate motion-blurred faces. *arXiv preprint arXiv:2112.07599* (2021).

19. Cossairt, O., Gupta, M. & Nayar, S. K. When does computational imaging improve performance? *IEEE Transactions on Image Process.* **22**, 447–458, DOI: 10.1109/TIP.2012.2216538 (2013).

20. Mait, J. N., Euliss, G. W. & Athale, R. A. Computational imaging. *Adv. Opt. Photon.* **10**, 409–483, DOI: 10.1364/AOP.10.000409 (2018).

21. Gu, J., Hitomi, Y., Mitsunaga, T. & Nayar, S. Coded rolling shutter photography: Flexible space-time sampling. In *2010 IEEE International Conference on Computational Photography (ICCP)*, 1–8, DOI: 10.1109/ICCPHOT.2010.5585094 (2010).

22. Elmalem, S., Giryes, R. & Marom, E. Motion deblurring using spatiotemporal phase aperture coding. *Optica* **7**, 1332–1340, DOI: 10.1364/OPTICA.399533 (2020).

23. Wei, M. *et al.* Coded two-bucket cameras for computer vision. In Ferrari, V., Hebert, M., Sminchisescu, C. & Weiss, Y. (eds.) *Computer Vision – ECCV 2018*, 55–73 (Springer International Publishing, Cham, 2018).

24. Antipa, N., Oare, P., Bostan, E., Ng, R. & Waller, L. Video from stills: Lensless imaging with rolling shutter. In *IEEE International Conference on Computational Photography (ICCP)*, 1–8 (2019).

25. Jin, M., Hu, Z. & Favaro, P. Learning to extract flawless slow motion from blurry videos. In *IEEE/CVF Conference on Computer Vision and Pattern Recognition (CVPR)* (2019).

26. Zhang, Y., Wang, C. & Tao, D. Video frame interpolation without temporal priors. In Larochelle, H., Ranzato, M., Hadsell, R., Balcan, M. & Lin, H. (eds.) *Advances in Neural Information Processing Systems*, vol. 33, 13308–13318 (Curran Associates, Inc., 2020).

27. Shen, W. *et al.* Blurry video frame interpolation. In *Proceedings of the IEEE/CVF Conference on Computer Vision and Pattern Recognition (CVPR)* (2020).

28. Holloway, J., Sankaranarayanan, A. C., Veeraraghavan, A. & Tambe, S. Flutter shutter video camera for compressive sensing of videos. In *IEEE International Conference on Computational Photography (ICCP)*, 1–9, DOI: 10.1109/ICCPhot.2012.6215211 (2012).

29. Liu, D. *et al.* Efficient space-time sampling with pixel-wise coded exposure for high-speed imaging. *IEEE Transactions on Pattern Analysis Mach. Intell.* **36**, 248–260, DOI: 10.1109/TPAMI.2013.129 (2014).

30. Llull, P. *et al.* Coded aperture compressive temporal imaging. *Opt. Express* **21**, 10526–10545, DOI: 10.1364/OE.21.010526 (2013).

31. Lin, S. *et al.* Learning event-driven video deblurring and interpolation. In Vedaldi, A., Bischof, H., Brox, T. & Frahm, J.-M. (eds.) *Computer Vision – ECCV 2020*, 695–710 (Springer International Publishing, Cham, 2020).

32. Barbastathis, G., Ozcan, A. & Situ, G. On the use of deep learning for computational imaging. *Optica* **6**, 921–943 (2019).

33. Wetzstein, G. *et al.* Inference in artificial intelligence with deep optics and photonics. *Nature* **588 7836**, 39–47 (2020).

34. Elmalem, S., Giryes, R. & Marom, E. Learned phase coded aperture for the benefit of depth of field extension. *Opt. Express* **26**, 15316–15331, DOI: 10.1364/OE.26.015316 (2018).

35. Sitzmann, V. *et al.* End-to-end optimization of optics and image processing for achromatic extended depth of field and super-resolution imaging. *ACM Trans. Graph.* **37**, DOI: 10.1145/3197517.3201333 (2018).

36. Akpinar, U., Sahin, E., Meem, M., Menon, R. & Gotchev, A. Learning wavefront coding for extended depth of field imaging. *IEEE Transactions on Image Process.* **30**, 3307–3320, DOI: 10.1109/TIP.2021.3060166 (2021).

37. Tan, S., Wu, Y., Yu, S.-I. & Veeraraghavan, A. Codedstereo: Learned phase masks for large depth-of-field stereo. *2021 IEEE/CVF Conf. on Comput. Vis. Pattern Recognit. (CVPR)* 7166–7175 (2021).

38. Haim, H., Elmalem, S., Giryes, R., Bronstein, A. M. & Marom, E. Depth estimation from a single image using deep learned phase coded mask. *IEEE Transactions on Comput. Imaging* **4**, 298–310, DOI: 10.1109/TCI.2018.2849326 (2018).

39. Wu, Y., Boominathan, V., Chen, H., Sankaranarayanan, A. & Veeraraghavan, A. Phasecam3d — learning phase masks for passive single view depth estimation. In *2019 IEEE International Conference on Computational Photography (ICCP)*, 1–12, DOI: 10.1109/ICCPHOT.2019.8747330 (2019).

40. Chang, J. & Wetzstein, G. Deep optics for monocular depth estimation and 3d object detection. In *ICCV* (2019).

41. Chen, W., Mirdehghan, P., Fidler, S. & Kutulakos, K. N. Auto-tuning structured light by optical stochastic gradient descent. *IEEE/CVF Conf. on Comput. Vis. Pattern Recognit. (CVPR)* 5969–5979 (2020).

42. Metzler, C., Ikoma, H., Peng, Y. & Wetzstein, G. Deep optics for single-shot high-dynamic-range imaging. In *CVPR* (2020).

43. Sun, Q., Tseng, E., Fu, Q., Heidrich, W. & Heide, F. Learning rank-1 diffractive optics for single-shot high dynamic range imaging. In *The IEEE Conference on Computer Vision and Pattern Recognition (CVPR)* (2020).

44. Tseng, E. *et al.* Differentiable compound optics and processing pipeline optimization for end-to-end camera design. *ACM Transactions on Graph.* **40**, 1–19, DOI: 10.1145/3446791 (2021).

45. Sun, Q., Wang, C., Fu, Q., Dun, X. & Heidrich, W. End-to-end complex lens design with differentiate ray tracing. *ACM Trans. Graph.* **40**, DOI: 10.1145/3450626.3459674 (2021).

46. Sun, Q. *et al.* End-to-end learned, optically coded super-resolution spad camera. *ACM Trans. Graph.* **39**, DOI: 10.1145/3372261 (2020).

47. Sheinin, M., O'Toole, M. & Narasimhan, S. G. Deconvolving diffraction for fast imaging of sparse scenes. In *Proc. ICCP* (IEEE, 2021).

48. Peng, Y. *et al.* Learned large field-of-view imaging with thin-plate optics. *ACM Trans. Graph.* **38**, DOI: 10.1145/3355089.3356526 (2019).

49. Dowski, E. R. & Johnson, G. E. Wavefront coding: a modern method of achieving high-performance and/or low-cost imaging systems. In *Optics & Photonics* (1999).

50. Robinson, M. D. & Stork, D. G. Joint design of lens systems and digital image processing. In *International Optical Design*, WB4, DOI: 10.1364/IODC.2006.WB4 (Optica Publishing Group, 2006).

51. Kellman, M. R., Bostan, E., Repina, N. A. & Waller, L. Physics-based learned design: Optimized coded-illumination for quantitative phase imaging. *IEEE Transactions on Comput. Imaging* **5**, 344–353 (2019).

52. Yanny, K. *et al.* Miniscope3d: optimized single-shot miniature 3d fluorescence microscopy. *Light. Sci. & Appl.* **9** (2020).

53. Nehme, E. *et al.* Learning optimal wavefront shaping for multi-channel imaging. *IEEE Transactions on Pattern Analysis Mach. Intell.* **43**, 2179–2192, DOI: 10.1109/TPAMI.2021.3076873 (2021).

54. Shechtman, Y. Recent advances in point spread function engineering and related computational microscopy approaches: from one viewpoint. *Biophys. reviews* (2020).

55. Goodman, J. W. *Introduction to Fourier Optics* (Roberts and Company Publishers, 1996).

56. Tran, D. *et al.* A closer look at spatiotemporal convolutions for action recognition. *2018 IEEE/CVF Conf. on Comput. Vis. Pattern Recognit.* 6450–6459 (2018).

57. Arduino. Arduino Nano. https://www.arduino.cc/en/pmwiki.php?n=Main/ArduinoBoardNano (Nano).

58. Nah, S. *et al.* Ntire 2019 challenge on video deblurring and super-resolution: Dataset and study. In *Proceedings of the IEEE/CVF Conference on Computer Vision and Pattern Recognition (CVPR) Workshops* (2019).

59. Ronneberger, O., Fischer, P. & Brox, T. U-net: Convolutional networks for biomedical image segmentation. In Navab, N., Hornegger, J., Wells, W. M. & Frangi, A. F. (eds.) *Medical Image Computing and Computer-Assisted Intervention – MICCAI 2015*, 234–241 (Springer International Publishing, Cham, 2015).

60. Sandler, M., Howard, A., Zhu, M., Zhmoginov, A. & Chen, L.-C. Mobilenetv2: Inverted residuals and linear bottlenecks. In *IEEE Conference on Computer Vision and Pattern Recognition (CVPR)* (2018).

61. Su, S. *et al.* Deep video deblurring for hand-held cameras. In *Proceedings of the IEEE Conference on Computer Vision and Pattern Recognition*, 1279–1288 (2017).

62. Bao, W. *et al.* Depth-aware video frame interpolation. In *IEEE Conference on Computer Vision and Pattern Recognition* (2019).
