# Supplementary figures and images for: Video reconstruction from a single motion blurred image using learned dynamic phase coding
Source: Sci Rep. 2023 Aug 21;13:13625. doi: 10.1038/s41598-023-40297-0 (PMC10442388; doi:10.1038/s41598-023-40297-0)

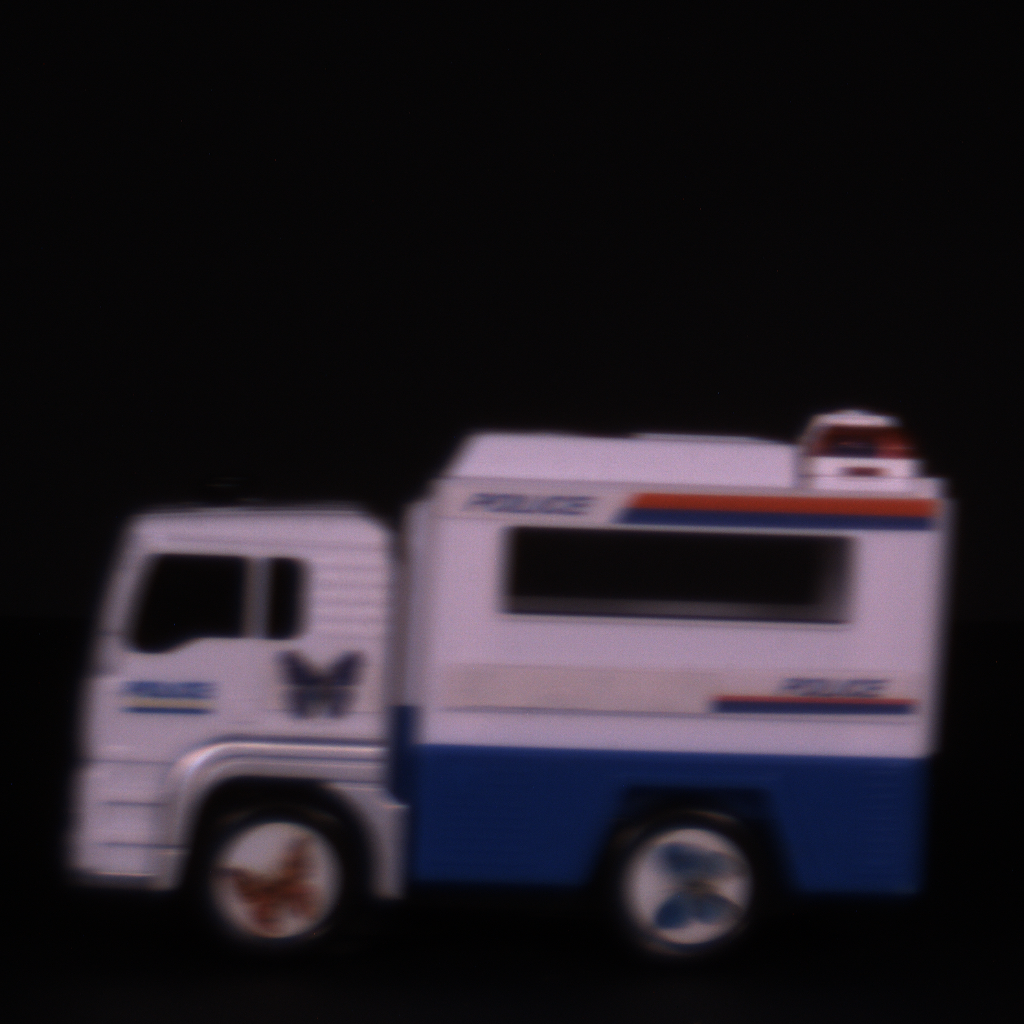

Supplement: Supplementary file 2 — Supplementary Information 2. [file 41598_2023_40297_MOESM2_ESM.zip › image-to-video demo code/car.png]

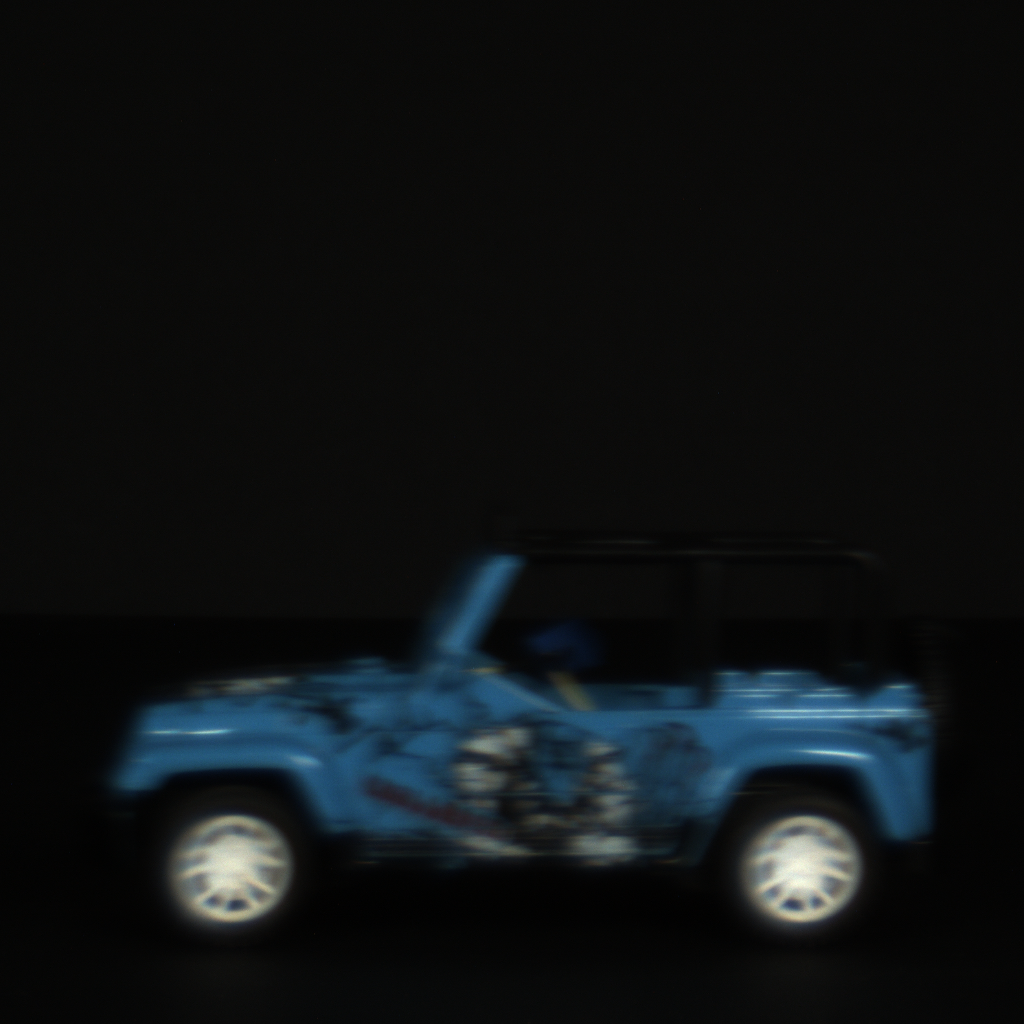

Supplement: Supplementary file 2 — Supplementary Information 2. [file 41598_2023_40297_MOESM2_ESM.zip › image-to-video demo code/car2.png]

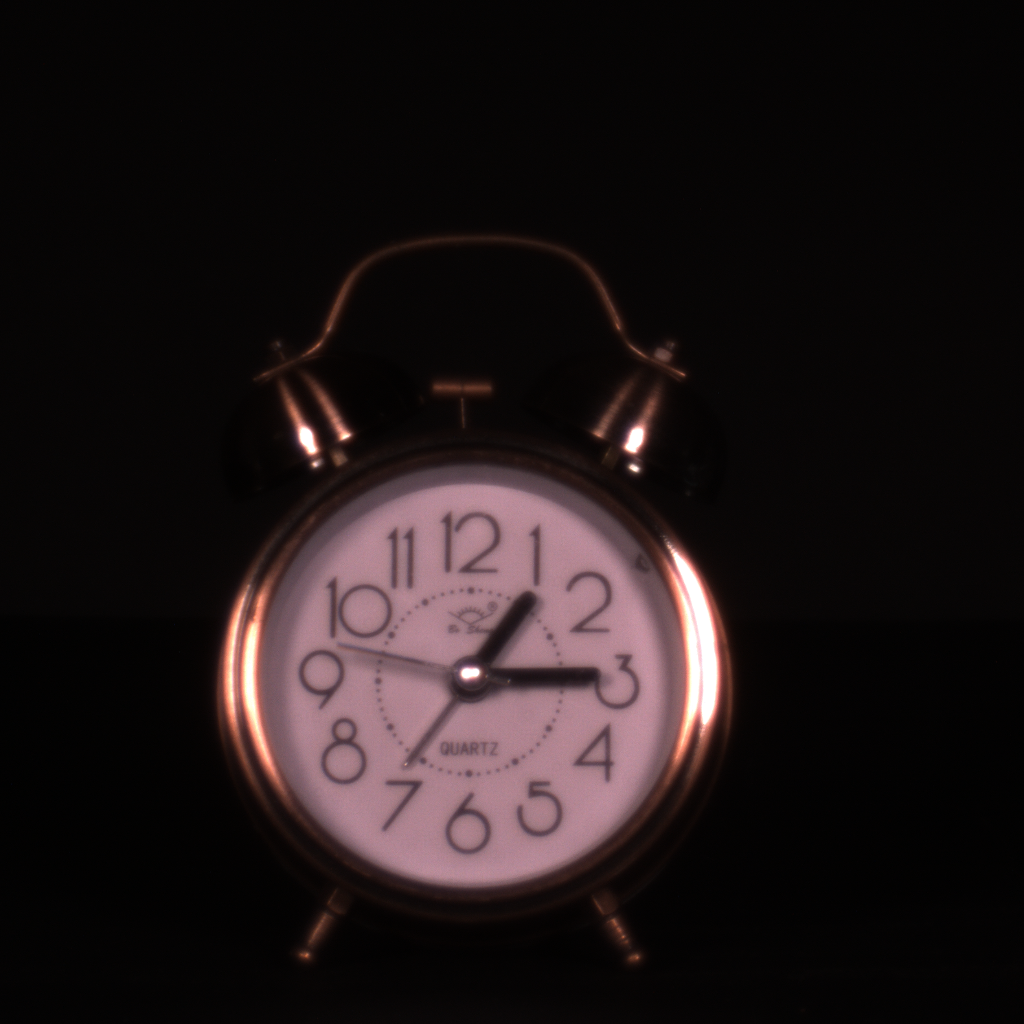

Supplement: Supplementary file 2 — Supplementary Information 2. [file 41598_2023_40297_MOESM2_ESM.zip › image-to-video demo code/clock.png]

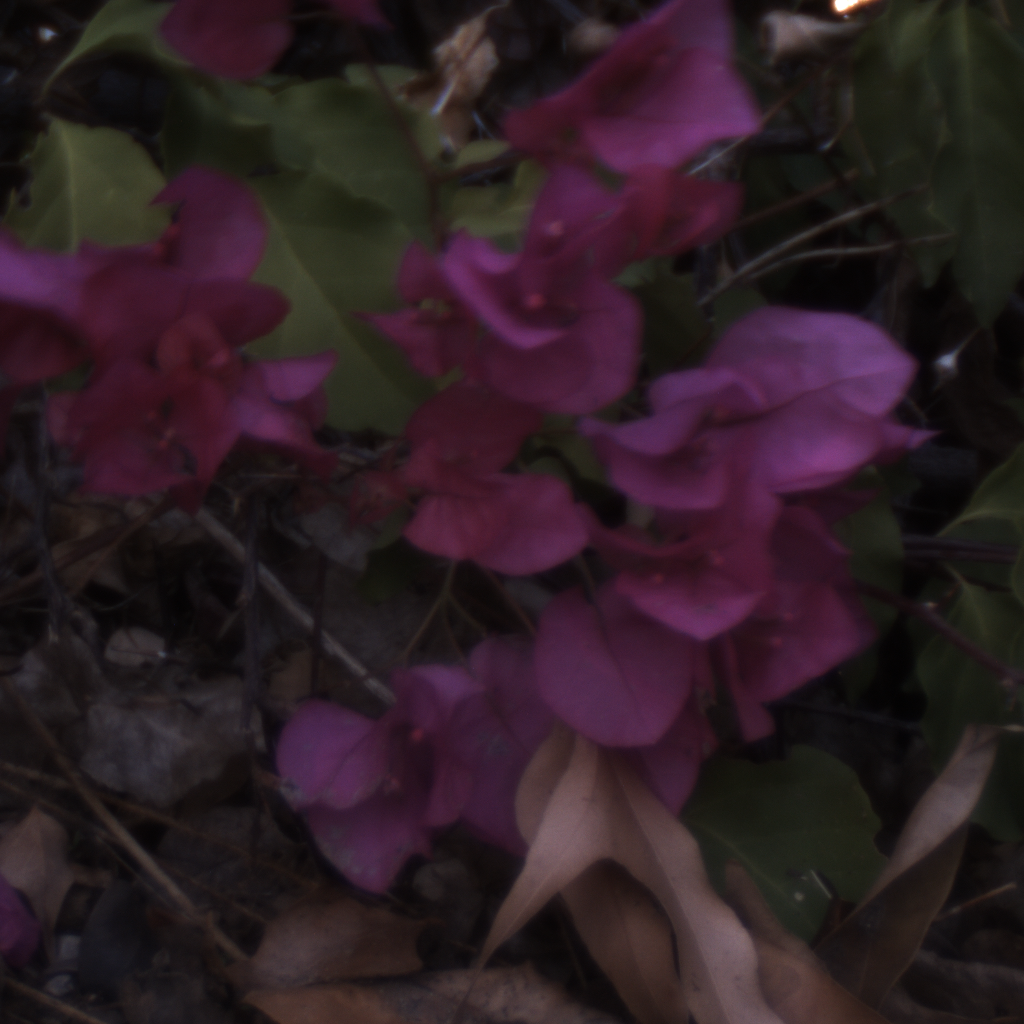

Supplement: Supplementary file 2 — Supplementary Information 2. [file 41598_2023_40297_MOESM2_ESM.zip › image-to-video demo code/out1.png]

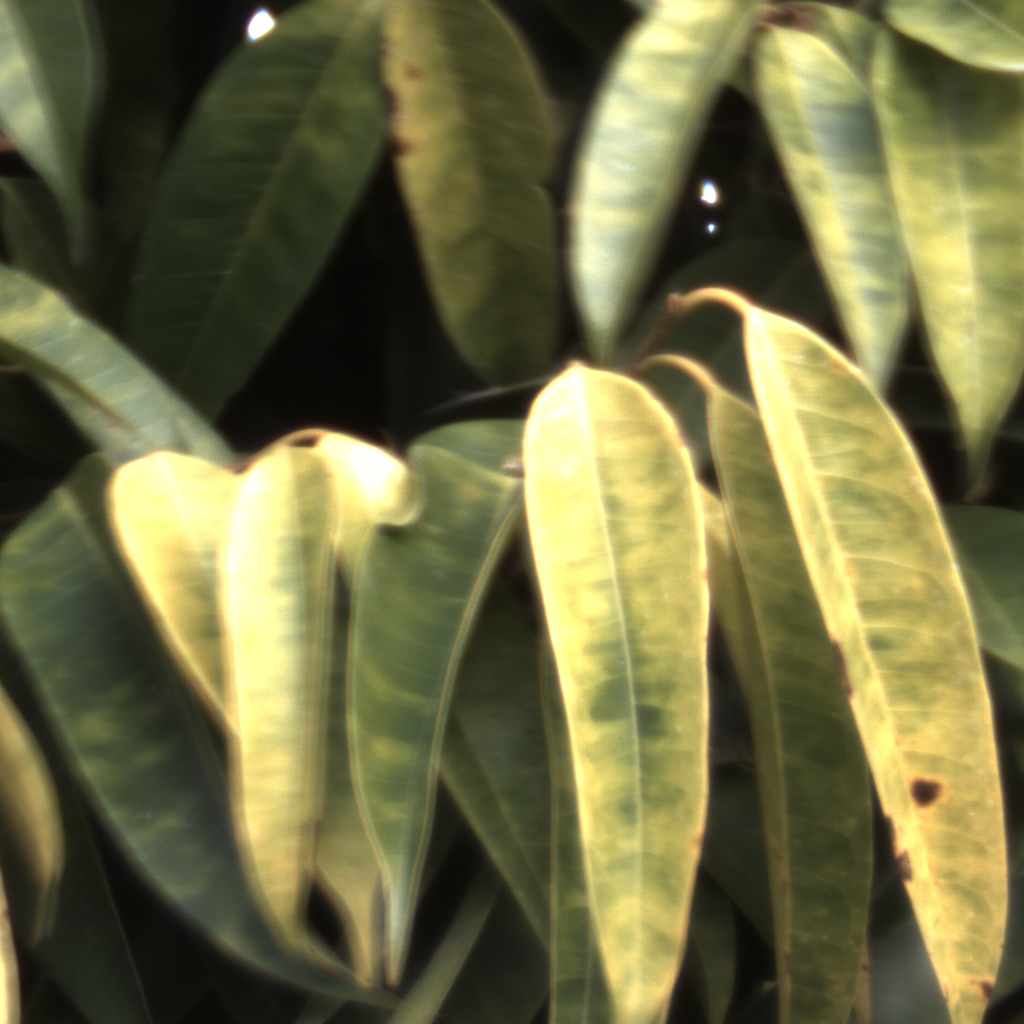

Supplement: Supplementary file 2 — Supplementary Information 2. [file 41598_2023_40297_MOESM2_ESM.zip › image-to-video demo code/out2.png]

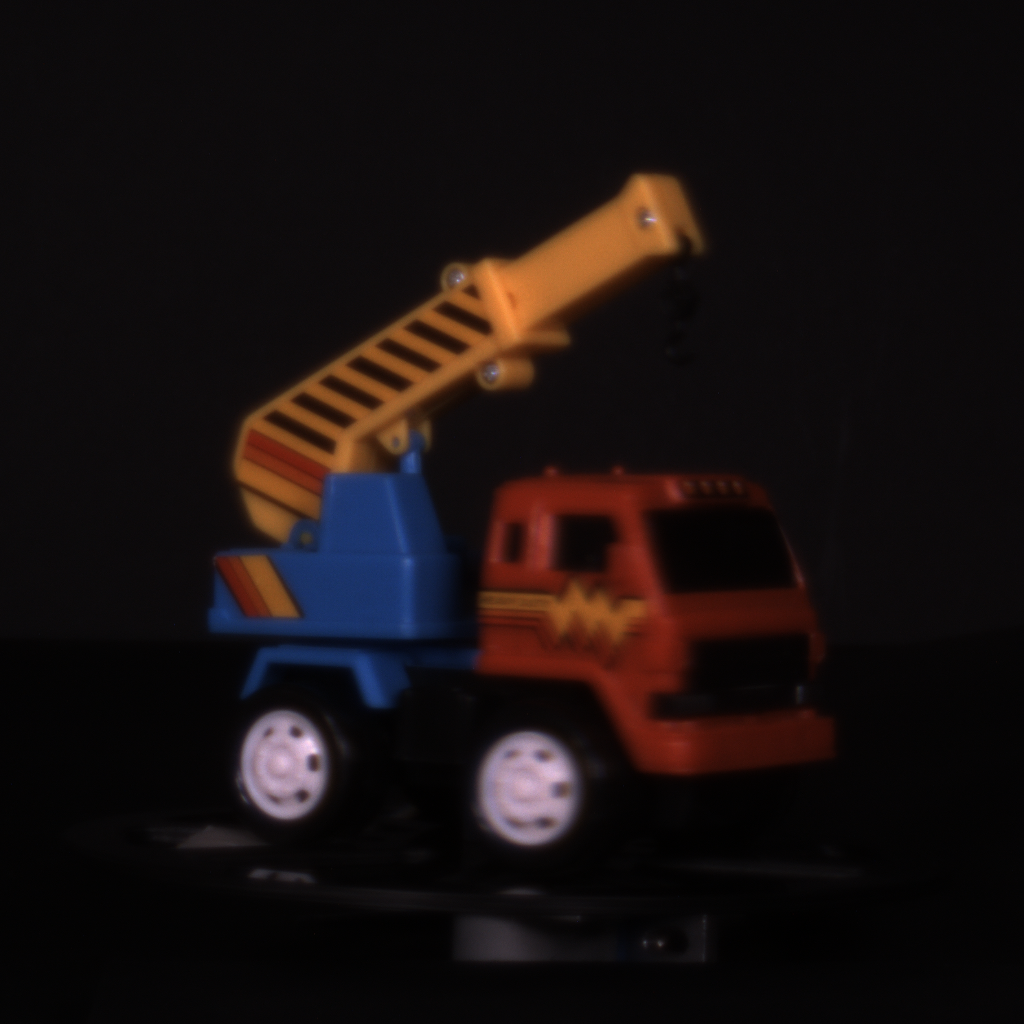

Supplement: Supplementary file 2 — Supplementary Information 2. [file 41598_2023_40297_MOESM2_ESM.zip › image-to-video demo code/rotcar_demo.png]

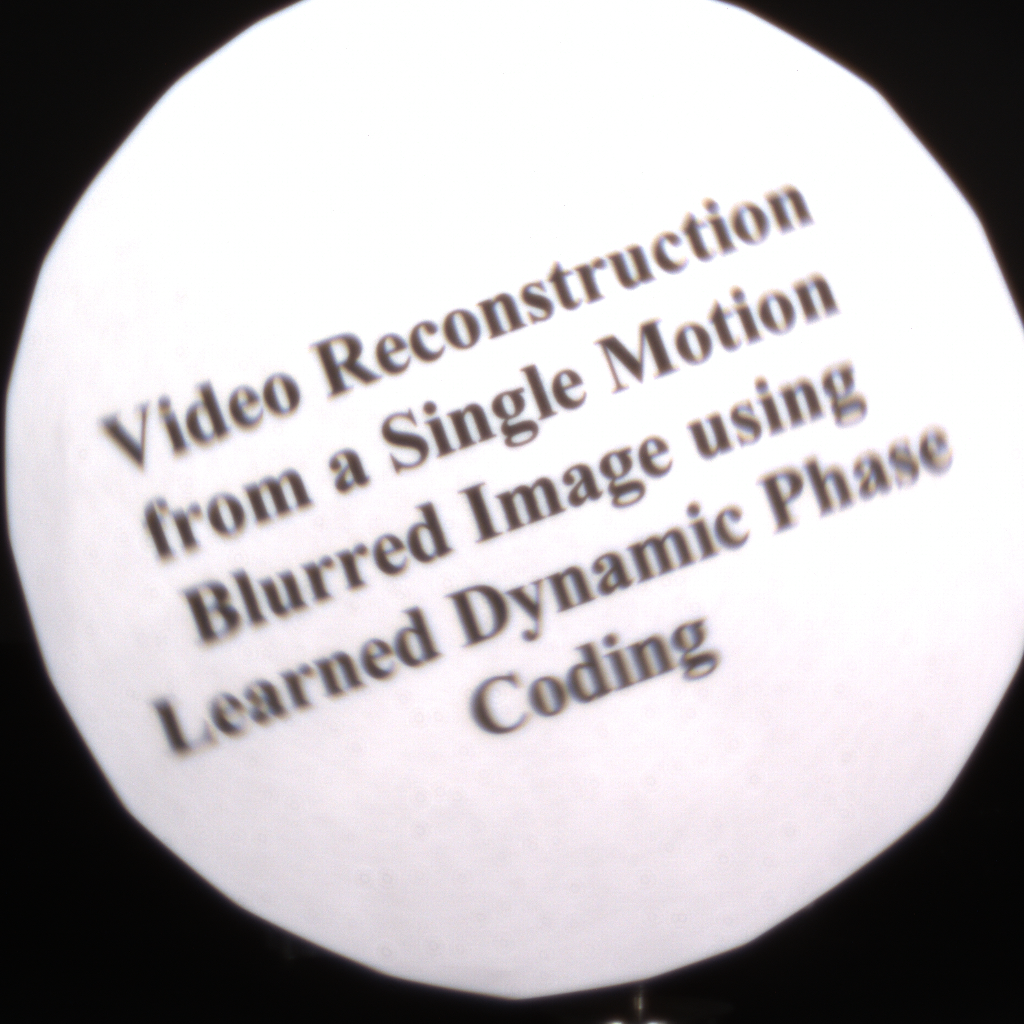

Supplement: Supplementary file 2 — Supplementary Information 2. [file 41598_2023_40297_MOESM2_ESM.zip › image-to-video demo code/title_demo.png]
